# Global trends in colorectal cancer and metabolic syndrome research: a bibliometric and visualization analysis

**DOI:** 10.1097/JS9.0000000000001342

**Published:** 2024-03-18

**Authors:** Peng-Ning Wu, Jia-Li Liu, Mei-Juan Fang, Xiao-Shuo Fu, Jia-Li Wei, Yue Wang, Hai-Hua Qian, Dan Zhang

**Affiliations:** Department of Anorectal Surgery, The Affiliated Hospital of Nanjing University of Chinese Medicine, Nanjing, People’s Republic of China

**Keywords:** bibliometrics, citespace, colorectal cancer, metabolic syndrome, VOSviewer

## Abstract

Numerous studies have demonstrated a robust correlation between metabolic syndrome (MetS) and colorectal cancer (CRC). Nonetheless, no systematic analysis or visualization of relevant publications has been conducted via bibliometrics. This research, centred on 616 publications obtainable through the Web of Science Core Collection (WoSCC), employed CiteSpace software and VOSviewer software for correlation analyses of authors, journals, institutions, countries, keywords, and citations. The findings indicate that the Public Library of Science had the highest number of publications, while the United States, China, and South Korea were the most contributory nations. Recent years have seen the mechanisms linking Metabolic Syndrome with Colorectal Cancer, including diet, obesity, insulin resistance, and intestinal flora, remain a burgeoning research area. Furthermore, bariatric surgery appears to be a promising new area of study. This paper presents the initial bibliometric and visualization analysis of research literature concerning CRC and MetS which examines research trends and hotspots.

## Introduction

Highlights

- This study provides the first bibliometric analysis and visualization of the field related to CRC and MetS.
- This research discovered that, besides CRC, MetS is strongly associated with several other cancer types, such as breast, prostate, liver, and pancreatic cancers.
- This study prompts that the mechanisms associated with MetS and CRC may continue to be a research hotspot, such as diet, obesity, insulin resistance, and intestinal flora. In addition, bariatric surgery may become a new hot spot.
- This study revealed that the United States, China, and South Korea are at the forefront in terms of published articles, researchers, and institutions, and hold robust connections with other nations.

CRC is the third most prevalent cancer in the world and, at the same time, it is one of the major treatable cancers^1,2^. MetS is an abnormality of metabolism including obesity, hypertension, insulin resistance, dyslipidemia and hyperglycemia^3–5^. MetS has been linked with about 13% increased risk of CRC. Obesity is an established risk factor for CRC and affects almost all major pathways of colorectal carcinogenesis, although the underlying mechanisms have not been fully elucidated^6–8^. For every 5 kg/m^2^ increase in body-mass index (BMI), the risk of obesity-related cancers increases by 24% for men and 9% for women^9^. MetS and its components are strongly associated with developing CRC. There has been much interest in studying both, and a growing body of specialized literature is emerging. However, the relationship between CRC and MetS has not been systematically studied through bibliometric and visual analysis. In-depth bibliometric studies of publications, countries, institutions, journals, authors, and keywords are still necessary.

Bibliometric analysis involves using mathematical and statistical methods to quantitatively analyze bodies of knowledge within a given discipline. This enables the examination of research priorities and hotspots within a field of study, as well as the assessment of the scientific productivity of countries, institutions, and researchers^10^. As such, it plays a significant role in depicting the features and upcoming tendencies of the discipline. This study is aimed at a comprehensive and systematic review of the state of research in CRC and MetS worldwide for the period 2013–2022, filling the gap in the field where bibliometric analyses are currently unavailable. CiteSpace and VOSviewer offer visual gateways to the scientific publication literature. The analysis indicates that bariatric surgery could be a novel research direction for tackling MetS and CRC-associated ailments. However, the existing literature emphasizes the necessity for further research to fully comprehend the pathogenesis of CRC and to enhance therapy options for MetS.

## Method

### Data source and literature search strategy

Literature for this study came from the Web of Science Core Collection (wosCC), restricted to ‘English’ papers published between 1 January 2012 and 1 January 2021, with paper type restricted to ‘papers’. We searched with subject and free terms about CRC and MetS (see Tables S1. Entry Terms and S2. Search strategy in the Supplementary Information, Supplemental Digital Content 1, http://links.lww.com/JS9/C157). Ultimately, the exported data included ‘full record and citations’ and was in ‘plain text’ format. The search was conducted independently by two researchers on 12th September 2023.

### Software for bibliometric analysis and visualization analysis

The data analysis and visualization software employed in this study utilized Microsoft Office Excel 2016, CiteSpace.5.7.R5, and Vosviewer1.6.19. Specifically, Microsoft Office Excel 2016 was employed for publication trend statistics, data collation and related tables. CiteSpace 5.8 R5 was applied to analyze the number of publications by country, institution, and author, the intermediary centrality and frequency of keywords, keyword bursts, and plotted as a visual map. VOSviewer 1.6.19 was used to investigate journals, co-cited literature and highly cited literature. The previously published article describes the specific parameter settings and interpretation of results in CiteSpace^11^. The time slices for the study run from 2013 to 2022. Various node types, including author, institution, country, and keyword, were examined.

## Result

### Publishing trend analysis

**Figure 1:**
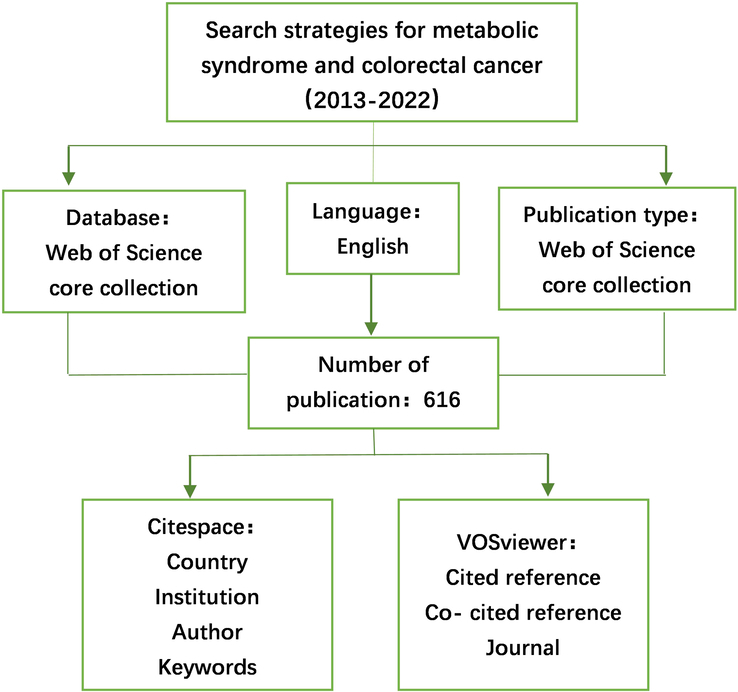
Flowchart of the study.

**Figure 2:**
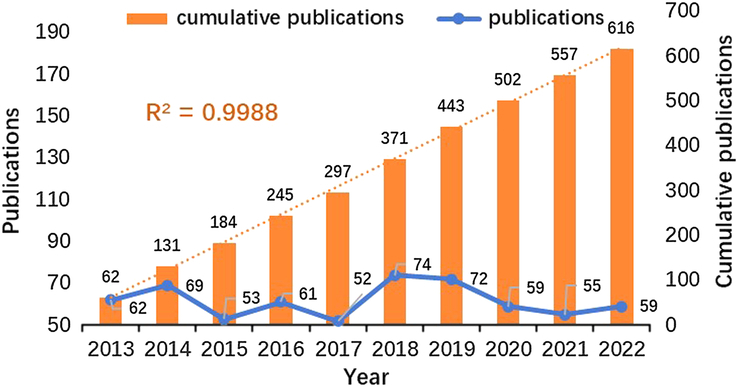
Published Trend Maps on CRC and MetS.

A total of 616 pertinent articles on CRC and MetS were identified in this study. The study’s specific flow is depicted in Fig. 1. Although the number of articles on CRC and MetS varied from high to low between 2013 and 2022, the overall trend is upward, and the cumulative number is steadily increasing, as indicated in Fig. 2. An exponential growth function was utilized to assess the correlation between cumulative publications and publication year, revealing alignment with the trend in cumulative publications (R^2^=0.998). This robust correlation indicates that research about CRC and MetS has considerably grown and progressed. Consequently, this denotes increasing interest in research regarding CRC and MetS in the preceding decade.

### Publications and collaborative networks of countries/regions, institutions and authors

We examined the quantity of publications and collaborative networks of countries, regions, institutions, and authors involved in CRC and MetS-related research. The larger the node in the graph, the greater the number of publications. The purple outer circle denotes a centrality value higher than 0.1 for the intermediary. Moreover, a larger centrality value indicates increased collaboration between the node and other nodes.

### Analysis of national publications and collaborations

**Figure 3:**
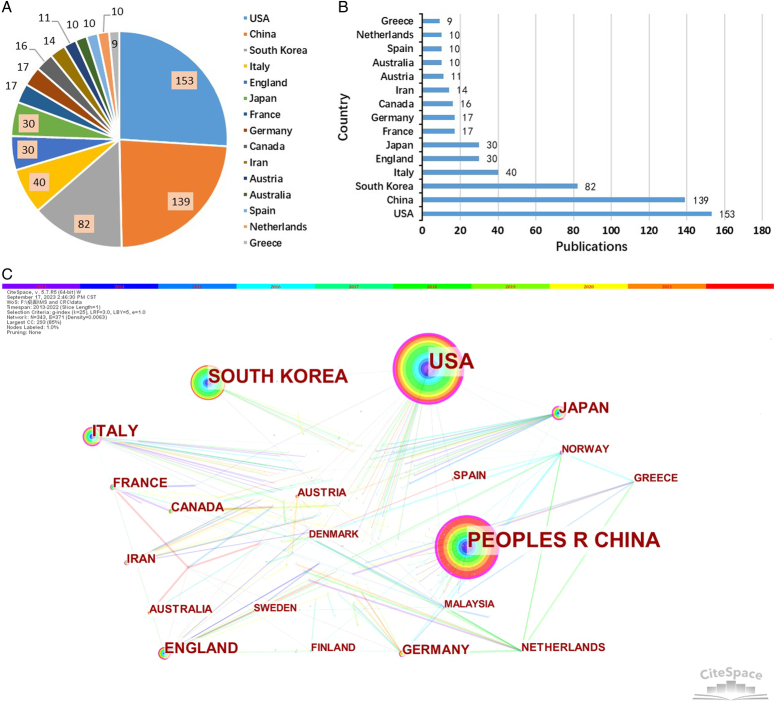
Each country/region’s contribution to the CRC and MetS. (A) Number of publications by country; (B) Intermediary centrality of countries; (C) collaborative networks of countries.

The study analyzed the publication count (Fig. 3A), mediator centrality (Fig. 3B), and collaborative networks (Fig. 3C) of CRC and MetS-related research in various countries/regions. Findings from Fig. 3A and B reveal that the United States, China, Korea, Italy, the United Kingdom, and Japan are the leading nations in publication count and mediator centrality. The United States came in first place with 153 publications, followed by China (139), South Korea (82), Italy (40), the United Kingdom (30), and Japan (30). The rest of the countries/regions had less than 20 publications. It is demonstrated that these six nations are the foremost power in the area of research on CRC and MetS. Furthermore, there is a robust correlation between each country. Specific country information about publication quantity and significance can be found in Table S3 of the Supplementary Information, Supplemental Digital Content 1, http://links.lww.com/JS9/C157.

### Analysis of institutional publications and collaborations

**Figure 4:**
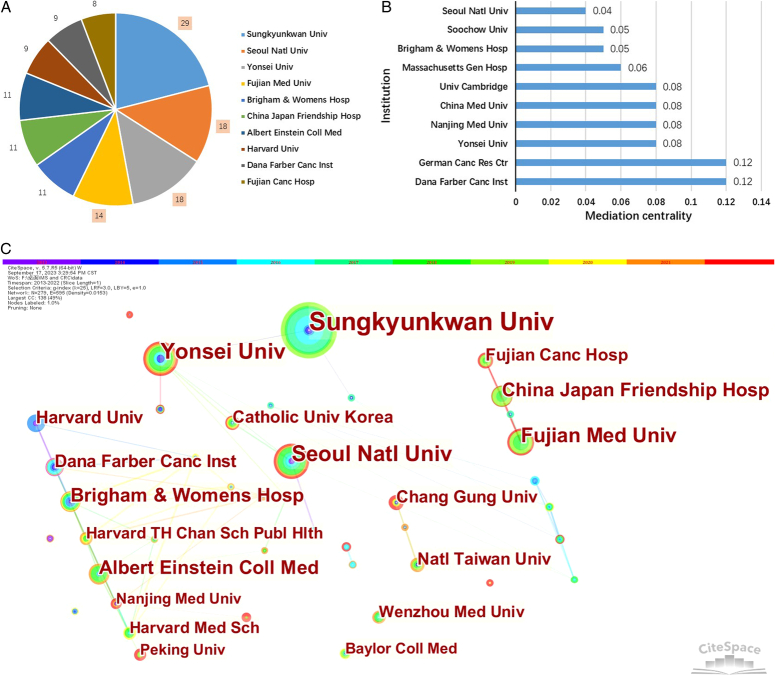
Each institution’s contribution to the CRC and MetS. (A) Number of publications by institution; (B) Intermediary centrality of institutions; (C) collaborative networks of institutions.

Fig. 4A displays publications from institutions, while Fig. 4B demonstrates the significance of institutions acting as intermediaries. Figure 4C illustrates a network diagram depicting collaboration between institutions. It is evident from this figure that the majority of total publications are contributed by four institutions namely Sungkyunkwan University, Seoul National University, Yonsei University, and Fujian Medical University. Furthermore, the centrality of intermediaries such as Dana-Farber Cancer Institute and German Cancer Research Center is greater than 0.1. Specific information regarding the quantity of published works and the significance of institutions can be located in Table S4 in the Supplementary Information, Supplemental Digital Content 1, http://links.lww.com/JS9/C157.

### Analysis of journal publication numbers and impact

Table 1 presents the top 20 journals based on publications exceeding 5 and their 2023 impact factors (IF). Metabolism-Clinical and Experimental (IF 9.8), the British Journal of Cancer (IF 8.8), and the International Journal of Cancer (IF 6.4) are among the top quartile (Q1) journals according to the Journal Citation Reports (JCR).

**Table 1:** Top 15 journals with the highest number of publications.

Rank	Journal	IF (2023)	JCR-C	Article
1	Metabolism-clinical and experimental	9.8	Q1	6
2	British journal of cancer	8.8	Q1	5
3	International journal of cancer	6.4	Q1	14
4	Nutrients	5.9	Q2	8
5	International journal of molecular sciences	5.6	Q2	6
6	Frontiers in oncology	4.7	Q3	9
7	Scientific reports	4.6	Q3	9
8	World journal of gastroenterology	4.3	Q2	11
9	Journal of gastroenterology and hepatology	4.1	Q3	11
10	Journal of cancer	3.9	Q3	9
11	Cancer epidemiology biomarkers and prevention	3.8	Q2	7
12	Bmc cancer	3.8	Q2	6
13	Public Library of Science	3.7	Q2	20
14	Digestive diseases and sciences	3.1	Q3	12
15	Bmj open	2.9	Q3	8

Note: According to the statistics, the journal ‘Oncotarget’ published 9 related papers. But in 2018, this journal was removed from the SCI (Science Citation Index) scope of coverage.

### Analysis of publications and cooperation among Authors

**Figure 5:**
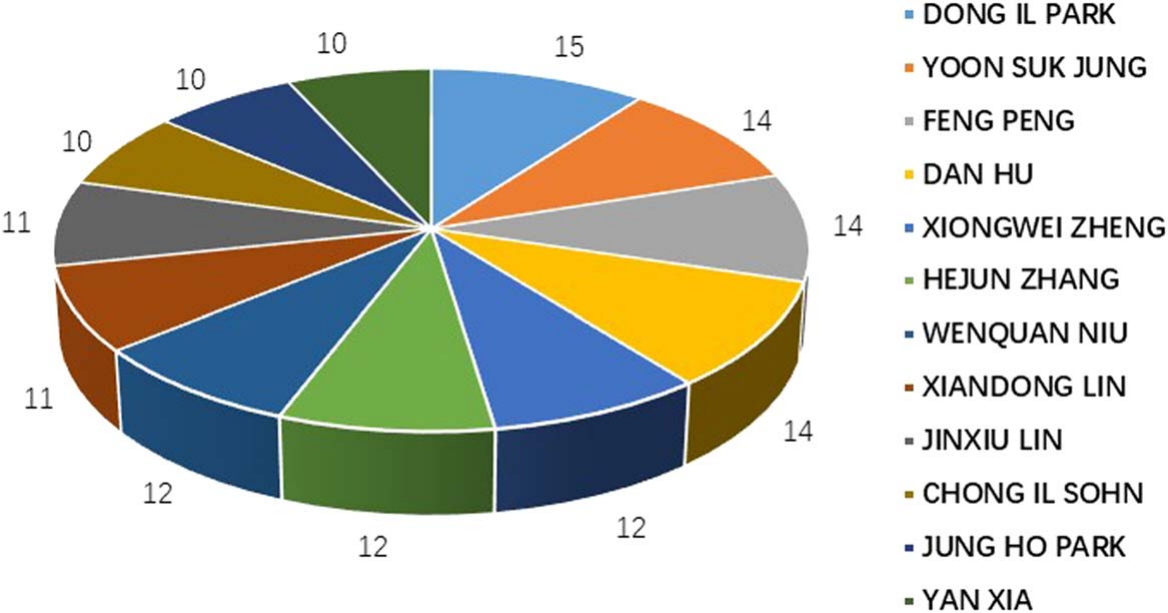
Each Author’s contribution to the CRC and MetS.

**Figure 6:**
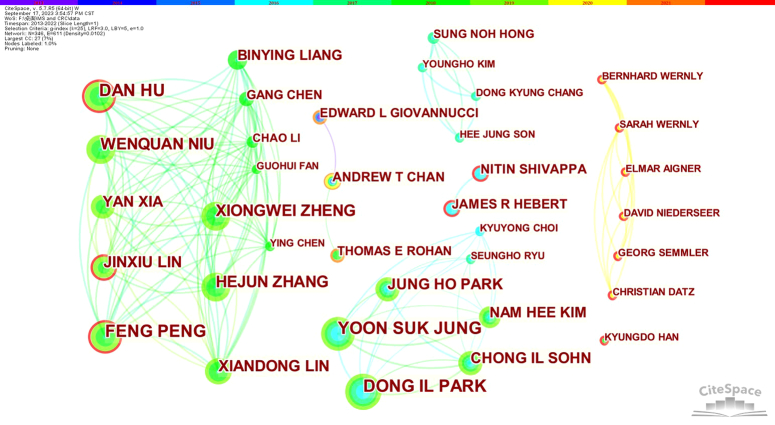
Authors’ collaboration on CRC and MetS.

We examined the quantity of published works and collaborative partnerships amongst authors investigating the link between CRC and MetS. As illustrated in Fig. 5, there were 12 authors with 10 or more publications. While there is collaboration amongst multiple authors with a significant number of publications, the level of cohesion of the collaboration may not be high, as illustrated in Fig. 6. Specific details on the number of publications and centrality can be found in Table S5 in the Supplementary Information, Supplemental Digital Content 1, http://links.lww.com/JS9/C157. Collaboration amongst such individuals may be related to a common interest in the field and the substantial financial support provided to the investigators.

### Research hot spots and trend analysis

#### Analysis of highly co-cited references

**Figure 7:**
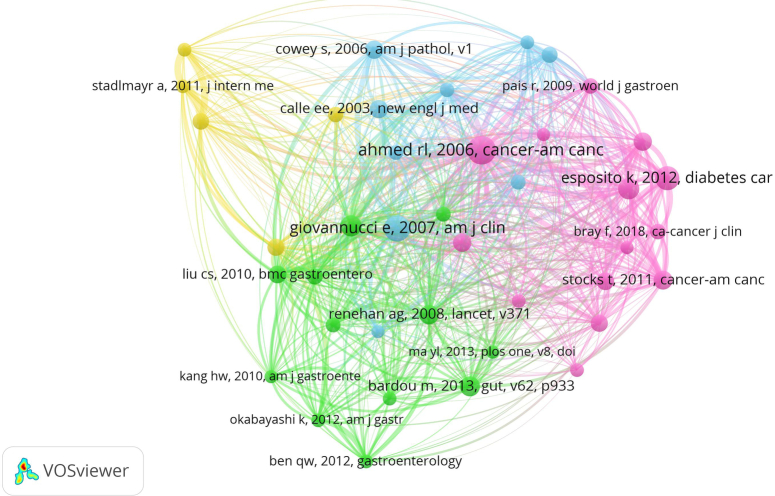
Cluster mapping of highly co-cited literature.

We employed VOSviewer to plot the co-cited references, revealing that a total of 23,114 references had been cited. When setting the minimum citation time to 20, the resulting number of documents in the analysis was reduced to 40. Figure 7 displays the final relationship graph. The highly co-cited references in the network graph can be categorized into four clusters, each represented by a different colour: pink, green, blue, and yellow. The specific literature clustering data is included in the Supplementary Information Table S6. Co-Citation information, Supplemental Digital Content 1, http://links.lww.com/JS9/C157. We have compiled a list of the top 10 most frequently cited papers, all with over 30 citations, as presented in the Supplementary Information Table S7. References, Supplemental Digital Content 1, http://links.lww.com/JS9/C157. The article with the highest number of citations was published in 2006 and is titled ‘The Metabolic Syndrome and Risk of Incident Colorectal Cancer’^12^. Following this, the second most cited paper was a review by Giovannucci E., named ‘Metabolic syndrome, hyperinsulinemia, and colon cancer: a review’^13^.

#### Analysis of highly cited references

Highly cited literature demonstrates its academic worth and professional significance. We conducted an analysis on the top 10 most cited works relevant to CRC and MetS research, all of which were cited over 200 times. The two most cited pieces were both published in the ‘Gut’ journal, as indicated in the Supplementary Information Table S7, Supplemental Digital Content 1, http://links.lww.com/JS9/C157. The top 10 highly cited references and co-cited references. One of the articles, ‘Obesity and colorectal cancer,’ was published by Bardou M *et al.* in 2013. This publication discusses the link between obesity and CRC. In 2016, Julian R Marchesi *et al.* published ‘The gut microbiota and host health: a new clinical frontier’, the most widely cited article with 1349 citations. The paper provides the latest findings in the field of gut diseases, examining the relationship of MetS and obesity-related diseases with liver disease, bowel disease, and CRC. These two articles together provide robust proof that MetS influences CRC.

#### Analysis of cited literature bursts

Citation burst reflects a large change in the citation frequency of a cited document in a given period. In 2018, Choi YJ *et al.* published an article in European Journal of Epidemiology titled ‘Abdominal obesity, glucose intolerance and decreased high-density lipoprotein cholesterol as components of the MetS are associated with the development of colorectal cancer.’^14^, which continues to be heavily cited from 2019 to 2022. As can be seen, the mechanisms by which the MetS affects cancer are becoming clearer after years of development. Specific literature information and graphics can be found in Supplementary Information Table S8. Citation Bursts, Supplemental Digital Content 1, http://links.lww.com/JS9/C157.

#### Analysis of keyword co-occurrence, burst, and cluster

High-frequency keywords indicate the current research trends in that field. The size of a node in the graph corresponds to the frequency of its keyword. The most frequent keywords from the map of co-occurrence of keywords and their detailed information are as follows: MetS, CRC, risk, obesity, association, cancer, insulin resistance, colon cancer, colorectal adenoma, mortality. Meanwhile, Table 2 provides specific keyword information on the following terms which ranked high for intermediate centrality: inflammation, prevalence, physical activity, survival, adipose tissue, fatty liver disease, smoking, prostate cancer, glucose, and oxidative stress.

**Table 2:** The top 20 keywords ranked by frequency and centrality.

Rank	Keywords	Frequency	Keywords	Centrality
1	Metabolic syndrome	413	inflammation	0.19
2	Colorectal cancer	292	prevalence	0.12
3	Risk	230	physical activity	0.11
4	Obesity	147	survival	0.1
5	Association	119	colon	0.09
6	Cancer	115	disease	0.09
7	Insulin resistance	108	adipose tissue	0.09
8	Colon cancer	94	fatty liver disease	0.09
9	Colorectal adenoma	77	smoking	0.09
10	Mortality	76	mortality	0.08
11	Body mass index	74	cardiovascular disease	0.08
12	Inflammation	66	prostate cancer	0.08
13	Metaanalysis	57	health	0.08
14	Prevalence	51	cohort	0.08
15	Breast cancer	51	diet	0.08
16	Survival	49	expression	0.07
17	Colon	43	colonoscopy	0.07
18	Physical activity	42	glucose	0.07
19	Cardiovascular disease	41	cohort study	0.07
20	Disease	39	oxidative stress	0.07

Furthermore, CiteSpace utilizes an algorithm to cluster keywords that are closely related. The higher the cluster ranking, the more keywords are incorporated into the cluster, and each cluster is denoted with keywords from the co-occurrence network. Specific information on the clustering of keywords can be located in Table S9. Keywords Cluster of the Supplementary Information, Supplemental Digital Content 1, http://links.lww.com/JS9/C157. A Silhouette value greater than 0.7 in the table confirms the validity of the clustering. The study identified the top ten clusters, namely adiponectin, gene expression, metabolism, diabetes, abdominal obesity, body mass index, epidemiology, vitamin D, probiotics, and prognosis.

Through keyword frequency analysis, we identified that the following cancers appeared more frequently: breast cancer (51 occurrences), prostate cancer (27 occurrences), liver cancer (19 occurrences), and pancreatic cancer (12 occurrences). Detailed data can be found in Supplementary Information Table S10. Keywords, Supplemental Digital Content 1, http://links.lww.com/JS9/C157.

## Discussion

### Publication trends and cooperation

Bibliometric methods were utilized in the analysis of the progression of research about CRC and MetS from 2013 to 2022. The research productivity within this domain has augmented due to the worldwide focus on MetS, the burden of cancer, and the evolving comprehension of its mechanisms. Widespread adoption of western lifestyles changes CRC incidence. Dietary patterns and the composition of diets have undergone dramatic changes over the past half century, with marked differences between regions of the world and within countries^15^. In Japan, for example, meat and fat intake increased steeply from the mid-1950s to the early to mid-1970s. During the period 1990–2000, there was a significant increase in colon cancer incidence and mortality rates^16^. Rapid changes in income and economic growth in low- and middle-income countries have altered dietary trends towards greater consumption of fats, sugars, and animal-based foods^17^. Simultaneously, changes in the food environment, including access to cheaper ‘junk’ foods, have been accompanied by reduced physical activity and increased sedentary behavior, increases in overweight and obesity, and changes in the built environment, all of which contribute to changing dietary patterns. In conclusion, the higher burden of CRC is largely attributable to Western lifestyles globally^18^. Therefore, it is crucial to enhance prevention efforts and treatments, particularly for patients with comorbid metabolic conditions. The augmentation of funding in research and rising support for research institutions in numerous countries have enabled effective collaboration between researchers, institutions, and countries, consequently contributing to the progressive enhancement of research and the rapid growth of the field.

### National publishing trends and cooperation

The top fifteen countries by publication count generated a total of 588 articles, representing 95.45% of the total output. The United States, China, and South Korea predominate among the fifteen countries in terms of publication count. Furthermore, China, the United States, and South Korea display the most robust international collaborations. The aforementioned findings establish the pivotal roles and authoritative positions of the United States, China, and South Korea in research on CRC and MetS. The United States, with its powerful economy, monolithic diet, and highly motivated researchers, is the most productive country in this field. China and South Korea exhibit high productivity in the field, potentially explained by the heightened incidence of CRC caused by westernized lifestyles as indicated in ‘Global patterns and trends in CRC incidence and mortality’^15^. Extensive international collaboration would greatly benefit the field in enhancing overall research quality.

### Institutional publishing trends and cooperation

Of the top ten organizations in terms of number of publications, four are from the United States, three are from Korea, and three are from China. The three institutions with the highest number of publications are all based in Korea, which aligns with the distribution of publications by country. Sungkyunkwan University, Seoul National University, Yonsei University, and Fujian Medical University comprise the majority of the published works. They are the principal drivers of research on MetS and CRC. Meanwhile, the Dana-Farber Cancer Institute and the German Cancer Research Center possess strong bonds and close cooperation with other establishments. This emphasizes the significance of seeking comprehensive partnerships among institutions, particularly under the influence of economic or resource limitations, to enhance research competitiveness.

### Journal publications volume and impact

Analyzing academic publications assists researchers in identifying suitable journals in their field for submitting articles. Peer-reviewed journals are essential for academic publishing. Public Library of Science had the highest number of publications with 20 papers and an impact factor of 3.7. In general, the journals that conducted more research on MetS and CRC were primarily classified as Q2-Q3. It is necessary to enhance the global impact of related journals.

### Research basics and hot spots

#### Tracing the research base from co-cited literature

Co-citations refer to the literature cited by researchers in common. The aim of performing co-citation analysis is to identify the research foundation of shared aspects between MetS and CRC. During the present study, highly co-cited literature was classified into four clusters utilizing VOSviewer. The pink cluster prominently contained meta-analyses, systematic evaluations and prospective cohort studies concentrating on MetS and CRC. A systematic evaluation discovered a possible association between MetS and risk of common cancers^19^. MetS is particularly a risk factor for CRC when it includes three of the following components: hypertension, increased waist circumference, hypertriglyceridemia, low levels of HDL cholesterol, or diabetes/hyperglycemia^12^.

Although epidemiological studies provide evidence of an association between obesity or diabetes and the risk of CRC, there is a lack of information regarding the relationship between MetS and colorectal adenomas. Therefore, the literature within the green cluster favours exploring the correlation between MetS and colorectal adenomas. Research has elucidated that abdominal obesity, dyslipidemia, and insulin resistance are influential factors among the components of MetS in developing colorectal adenoma^20–22^.

The blue cluster examines the effects of diverse components of the MetS on cancer. The yellow cluster investigates the correlation between non-alcoholic fatty liver disease (NALD) and colorectal adenomas as well as CRC. The investigation discovered that there was a high incidence of colorectal adenomas and advanced tumours linked with NAFLD. Right-sided colonic adenomas were more commonly found. And CRC screening is vital for this high-risk population^23,24^.

In 2006, Rehana L Ahmed and colleagues investigated the association between MetS and an increased risk of bowel cancer. They used data from the multicentre, prospective Atherosclerosis Risk in Communities (ARIC) cohort study. MetS was defined as having at least three of the following disorders: high blood pressure, enlarged waist circumference, hypertriglyceridemia, low HDL cholesterol levels or diabetes mellitus/hyperglycemia. It is noteworthy that the aforementioned investigation established that MetS engenders a CRC risk for men exclusively in the study population^12^. This study is foundational in paving the way for additional cohort studies and meta-analyses that examine the links between MetS, CRC, and colorectal adenomas.

In 2007, a review by Giovannucci E. achieved the second-highest number of co-citations. The review summarized a significant amount of epidemiological data to demonstrate that the incidence of colon cancer is higher among individuals with MetS. This conclusion is supported by research examining the relationship between colon cancer or adenoma risk and the determinants of MetS, including obesity, abdominal adipose distribution, and physical inactivity, as well as the clinical outcomes of this syndrome, such as type 2 diabetes and hypertension. The plasma or serum components that define MetS include hypertriglyceridemia, hyperglycemia, and low levels of HDL cholesterol. Additionally, markers of hyperinsulinemia or insulin resistance, specifically insulin and C-peptide, are indicative of the underlying metabolic defect in individuals with MetS. It is worth emphasizing that although the mechanisms underlying these associations are unclear, they may involve the effects of hyperinsulinemia on increasing free or bioavailable concentrations of insulin-like growth factor-1^13^. This research recommends that forthcoming studies could rely on gauges of insulin resistance, beta-cell reduction, and insulin retort to ascertain more precisely which factors of insulin resistance are most robustly affiliated with colon tumor risk.

#### Discovering research hotspots from highly cited literature

The aim of analyzing heavily cited literature is to identify the areas of research focus and trends in the shared domains of CRC and MetS. From our exploration of highly cited literature, we discovered that six out of the top ten publications were associated with intestinal flora. These findings imply that the shared research focus in the realm of CRC and MetS is closely linked to intestinal flora, which emerges as a crucial research topic.

In 2013, Bardou and colleagues summarized the mechanism linking obesity to CRC. While controversial, altered levels of adipocytokines, MetS, and insulin resistance appear to play a prominent role. Additionally, emerging evidence suggests a contribution from biological factors such as gut microbiota and bile acids^25^.

In 2016, Marchesi *et al.* ^26^ presented updated knowledge in the field of intestinal diseases, with a special focus on MetS and obesity-related diseases, liver diseases, and intestinal disorders. The manipulation potential of the gut microbiota in these diseases is evaluated via the analysis of the latest and most pertinent evidence related to antibiotics, probiotics, prebiotics, polyphenols, and fecal microbiota transplantation. As well as reflecting research trends, this article informs gut flora-based therapy for MetS -associated CRC. Over the same year, Kolb R *et al.* posited that obesity may impact cancer through an inflammatory response. The causal link between obesity and an increase in cancer incidence and related mortality has been extensively reported for the past twenty years. It is estimated that obesity contributes to 14% of cancer mortality in men and 20% in women. Chronic inflammation, a phenotype linked to obesity, is a significant factor in the progression of chronic diseases. Alongside CRC, obesity can increase the risk of developing several other cancers, such as breast, liver, and pancreatic cancer^25^.

In 2020, Mantovani A and colleagues highlighted the robust correlation between NAFLD, CRC, and MetS. NAFLD is linked to an increased risk of developing significant extrahepatic conditions, including cardiovascular disease (which is the primary cause of death in NAFLD patients), extrahepatic cancers (specifically CRC), type 2 diabetes mellitus, and chronic kidney disease^27^. This article indicates that the interaction between NAFLD, CRC, and MetS is a current research area of interest.

In summary, frontier research is focusing on the links between MetS and its different components with gut diseases, such as MetS affecting CRC through the gut flora. Additionally, individual components of MetS can affect liver and intestinal diseases through gut microbiota. This highlights the potential for therapeutic interventions targeted at manipulating gut microbiota^25,28,29^.

#### Analyzing research hotspots through literature burst analysis

Literature burst analysis enables the systematic exploration of a scientific field by identifying recent trends and hotspots. The present study reveals that the investigation of the effects and mechanisms of distinct factors of MetS on CRC is currently at the forefront of research in this field.

In 2015, Mendonça and colleagues elucidated the pathological mechanism that links MetS and its components to carcinogenesis, including CRC^30^. The mechanism involves various players, including insulin, the insulin-like growth system, estrogen, pro-inflammatory cytokines, and others. Insulin and insulin-like growth factors (IGF) can enhance the growth of colorectal tumors through their proliferative and anti-apoptotic effects^31^. Meanwhile, estrogen may play a role in the sex-specific differences involving MetS and cancer^14^. Estrogen directly regulates glucose and lipid metabolism, and if it is lacking or testosterone levels are relatively elevated, this can induce insulin resistance and a proatherogenic lipid profile^32^. Remarkably, this difference has led to variable results in some trials. Furthermore, MetS patients have reduced expression of adiponectin, which has anti-inflammatory effects^33^. Grasping these mechanisms has tremendous clinical and economic implications for healthcare systems worldwide, as cancer is one of the leading causes of death globally. Further research is required to establish how distinct metabolic components establish CRC sites, and rectifying MetS or specific components will forestall cancer development.

#### Exploring research hotspots through keyword co-occurrence and burst analysis

Similarly, research hotspots can be identified via keyword co-occurrence and burst analysis. The research hotspots related to CRC and MetS mainly focus on risk, colorectal adenoma, inflammation, insulin resistance, and obesity, according to the frequency of keyword occurrence. Mediating centrality data suggests that various factors are closely related to the study of CRC and MetS, including inflammation, prevalence, physical activity, survival, adipose tissue, fatty liver disease, smoking, prostate cancer, glucose, oxidative stress, and other domains. It is noteworthy that apart from CRC, additional cancers including breast cancer, prostate cancer, liver cancer, and pancreatic cancer may be closely related to MetS. Further analysis of the frequency of keywords relating to these cancers and MetS revealed a high occurrence of insulin resistance. This suggests that insulin resistance may be the underlying mechanism connecting these cancers. Recent Keyword burst analysis indicated a surge in terms such as metformin, hypertension, mechanism, blood pressure, and metabolism in the last five years, providing an insight into research trends. Finally, keyword clustering suggests that epidemiology, mechanism, diagnosis and treatment are all research priorities in studies related to CRC and MetS.

#### Searching the latest literature to validate and find new research hotspots

To determine the consistency of recent literature with previous research trends, a search was conducted for the latest published articles on CRC and MetS up until October 4, 2023. The search yielded 35 ARTICLES and 16 REVIEWS (Details can be viewed in the Table S11. new reference in the Supplementary Information, Supplemental Digital Content 1, http://links.lww.com/JS9/C157), It can be noticed that the current literature focuses on the mechanisms linking MetS and CRC, including diet (such as carbohydrates and dairy products), obesity, gut microbiota, and IGF^34–39^. Interestingly, a systematic review and meta-analysis by Wilson RB *et al.* published this year in the International Journal of Molecular Sciences has been cited 6 times, which elaborates on the fact that bariatric surgery may lead to long-term reduction of body weight and improvement of MetS in morbidly obese patients. Previous studies have shown that obesity and gut flora are both strongly associated with increased cancer risk and the formation of various tumors^40^. At the same time, many studies have demonstrated the interaction between obesity and gut flora^41–43^. Bariatric surgery can simultaneously treat obesity and regulate disturbed gut microbiota and metabolites^44–46^. Besides, bariatric surgery may reduce future cancer morbidity and mortality overall^47^. These findings have implications for future research directions.

### Limitations of the research

Our study has some limitations, akin to prior bibliometric studies^11^. Firstly, we selected the WOSCC database for our study, limiting our results to English language articles which may have led to some missing data from the literature. Secondly, there were variations in name formats for some authors or institutions in the WOSCC database, potentially resulting in a scattered number of studies. Lastly, this paper cannot guarantee that each publication obtained fully meets the search criteria regarding topic relevance. Nevertheless, our analysis provides adequate results to reflect the current state of the field.

## Conclusion

This study quantified CRC and MetS studies in the WOSCC database over a decade using bibliometric and visualization analyses. The six nations with the highest number of publications and intermediary centrality are the United States, China, South Korea, Italy, the United Kingdom, and Japan. In conclusion, the prominent research areas that overlap in CRC and MetS concentrate on the subsequent topics: 1. The gut microbiota is frequently linked to both MetS and CRC and manipulating the gut microbiota may therefore be a viable treatment for CRC by addressing MetS; 2. Chronic inflammation is closely associated with different aspects of MetS, including obesity; 3. NAFLD interacts with CRC and MetS; 4. The mechanisms underlying the interaction between MetS and CRC are closely linked to insulin and the insulin-like growth system, insulin resistance, oestrogen, inflammation, obesity, hypertension and metabolism. Future research in this area is warranted. A current and emerging trend may be metabolic syndrome-associated colorectal cancer through gut microbiota modulation and bariatric surgery. This timely review scrutinizes research trends and hotspots related to CRC and MetS, which could progress the field and form the basis for forthcoming studies.

## Ethical approval

As all analyses were based on a literature review, no ethical approval or patient consent was required.

## Consent

This disclosure does not apply to this study, which is based on literature.

## Sources of funding

This research was supported by the State Administration of Traditional Chinese Medicine of the People's Republic of China (JD2019SZXZD01). Thanks to the State Administration of Traditional Chinese Medicine of the People's Republic of China for its funding support.

## Author contribution

The study was conceived and designed by D.Z. and H-H.Q.; P-N.W. wrote the manuscript and participated in the study design; P-N.W., JL.L., M-J.F., and XS.F. were responsible for searching literature and analyzing data; J-L.W. and Y.W. performed data interpretation and map-making. All authors contributed to the article and reviewed the submitted version.

## Conflicts of interest disclosure

The authors affirm that the study was carried out without any commercial or financial ties that might be interpreted as a potential conflict of interest.

## Research registration unique identifying number (UIN)

As all analyses were based on a literature review, this criterion was not applicable to this study.

## Guarantor

Dan Zhang, Department of Anorectal Surgery, The Affiliated Hospital of Nanjing University of Chinese Medicine, Nanjing, Jiangsu 210029, China, E-mail: danaezhang@163.com. Hai-Hua Qian, Department of Anorectal Surgery, The Affiliated Hospital of Nanjing University of Chinese Medicine, Nanjing, Jiangsu 210029, China, E-mail: haihuaqian@126.com.

## Data availability statement

The data used in this study is not sensitive and can be accessed publicly. Therefore, it is available and not confidential in nature. All data used in the study have been included in the article and Supplementary Information.

## Provenance and peer review

Not applicable.

## Supplementary Material
