# Supplementary material for: Global trends in colorectal cancer and metabolic syndrome research: a bibliometric and visualization analysis
Source: Int J Surg. 2024 Mar 18;110(6):3723–33. doi: 10.1097/JS9.0000000000001342 (PMC11175816; doi:10.1097/JS9.0000000000001342)
Supplement: Supplementary file 1 [file js9-110-3723-s001.docx]

| 1.Entry Terms **The entry Terms of metabolic syndrome and colorectal neoplasms** | |
| --- | --- |
| **Item** | **Entry Terms** |
| Metabolic Syndrome | Metabolic Syndromes; Syndrome, Metabolic; Syndromes, Metabolic; Metabolic Syndrome X; Insulin Resistance Syndrome X; Syndrome X, Metabolic; Syndrome X, Insulin Resistance; Metabolic X Syndrome; Syndrome, Metabolic X; X Syndrome, Metabolic; Dysmetabolic Syndrome X; Syndrome X, Dysmetabolic;Reaven Syndrome X; Syndrome X, Reaven; Metabolic Cardiovascular Syndrome; Cardiovascular Syndrome, Metabolic; Cardiovascular Syndromes, Metabolic; Syndrome, Metabolic Cardiovascular; Cardiometabolic Syndrome; Cardiometabolic Syndromes; Syndrome, Cardiometabolic; Syndromes, Cardiometabolic |
| Colorectal Neoplasms | Colorectal Neoplasm; Neoplasm, Colorectal; Neoplasms, Colorectal; Colorectal Tumors; Colorectal Tumor; Tumor, Colorectal; Tumors, Colorectal; Colorectal Cancer; Cancer, Colorectal; Cancers, Colorectal; Colorectal Cancers; Colorectal Carcinoma; Carcinoma, Colorectal; Carcinomas, Colorectal; Colorectal Carcinomas |

# 2.Search strategy

| **Step** | **Contents** | **Result** |
| --- | --- | --- |
| #1 | [((((((((((((((((((((((TS=(metabolic syndrome)) OR TS=(Metabolic Syndromes)) OR TS=(Syndrome, Metabolic)) OR TS=(Syndromes, Metabolic)) OR TS=(Metabolic Syndrome X)) OR TS=(Insulin Resistance Syndrome X)) OR TS=(Syndrome X, Metabolic)) OR TS=(Syndrome X, Insulin Resistance)) OR TS=(Metabolic X Syndrome)) OR TS=(Syndrome, Metabolic X)) OR TS=(X Syndrome, Metabolic)) OR TS=(Dysmetabolic Syndrome X)) OR TS=(Syndrome X, Dysmetabolic)) OR TS=(reuven Syndrome X)) OR TS=(Syndrome X, reuven)) OR TS=(Metabolic Cardiovascular Syndrome)) OR TS=(Cardiovascular Syndrome, Metabolic)) OR TS=(Cardiovascular Syndromes, Metabolic)) OR TS=(Syndrome, Metabolic Cardiovascular)) OR TS=(Cardiometabolic Syndrome)) OR TS=(Cardiometabolic Syndromes)) OR TS=(Syndrome, Cardiometabolic)) OR TS=(Syndromes, Cardiometabolic)](https://www.webofscience.com/wos/woscc/summary/b6b887a3-a28e-423d-87a5-34ae50e44d44-a325fa02/relevance/1) | 132406 |
| #2 | ((((((((((((((TS=(Colorectal Neoplasm)) OR TS=(Neoplasm, Colorectal)) OR TS=(Neoplasms, Colorectal)) OR TS=(Colorectal Tumors)) OR TS=(Colorectal Tumor)) OR TS=(Tumor, Colorectal)) OR TS=(Tumors, Colorectal)) OR TS=(Colorectal Cancer)) OR TS=(Cancer, Colorectal)) OR TS=(Cancers, Colorectal)) OR TS=(Colorectal Cancers)) OR TS=(Colorectal Carcinoma)) OR TS=(Carcinoma, Colorectal)) OR TS=(Carcinomas, Colorectal)) OR TS=(Colorectal Carcinomas) | 229000 |
| #3 | #2 AND #1 | 1188 |
| #4 | ((DT=(Article)) AND DOP=(2013-01-01/2022-12-31)) AND LA=(English) | 16716532 |
| #5 | #3 AND #4 | 616 |

# 3.Country

| **Top 15 Country/Region ranked by publications and centrality** | | | | |
| --- | --- | --- | --- | --- |
| **Ranking** | **Country/Region** | **Article** | **Country/Region** | **Centrality** |
| 1 | USA | 153 | China | 0.68 |
| 2 | China | 139 | USA | 0.5 |
| 3 | South Korea | 82 | South Korea | 0.36 |
| 4 | Italy | 40 | England | 0.23 |
| 5 | England | 30 | Italy | 0.21 |
| 6 | Japan | 30 | Japan | 0.14 |
| 7 | France | 17 | Spain | 0.12 |
| 8 | Germany | 17 | Iran | 0.09 |
| 9 | Canada | 16 | Malaysia | 0.08 |
| 10 | Iran | 14 | Germany | 0.07 |
| 11 | Austria | 11 | Canada | 0.06 |
| 12 | Australia | 10 | Turkey | 0.06 |
| 13 | Spain | 10 | France | 0.05 |
| 14 | Netherlands | 10 | Austria | 0.05 |
| 15 | Greece | 9 | Sweden | 0.04 |

# 4.Institution

| **４.１The institutions ranked by publications and centrality** | | | | | | | | | |
| --- | --- | --- | --- | --- | --- | --- | --- | --- | --- |
| **Ranking** | | **Institutions** | **Articles** | | | **Institutions** | | **Centrality** | |
| 1 | | Sungkyunkwan Univ | 29 | | | Dana Farber Canc Inst | | 0.12 | |
| 2 | | Seoul Natl Univ | 18 | | | German Canc Res Ctr | | 0.12 | |
| 3 | | Yonsei Univ | 18 | | | Yonsei Univ | | 0.08 | |
| 4 | | Fujian Med Univ | 14 | | | Nanjing Med Univ | | 0.08 | |
| 5 | | Brigham & Womens Hosp | 11 | | | China Med Univ | | 0.08 | |
| 6 | | China Japan Friendship Hosp | 11 | | | Univ Cambridge | | 0.08 | |
| 7 | | Albert Einstein Coll Med | 11 | | | Massachusetts Gen Hosp | | 0.06 | |
| 8 | | Harvard Univ | 9 | | | Brigham & Womens Hosp | | 0.05 | |
| 9 | | Dana Farber Canc Inst | 9 | | | Soochow Univ | | 0.05 | |
| 10 | | Fujian Canc Hosp | 8 | | | Seoul Natl Univ | | 0.04 | |
| 11 | | Chang Gung Univ | 8 | | | Harvard Med Sch | | 0.04 | |
| 12 | | Catholic Univ Korea | 8 | | | Canc Registry Norway | | 0.04 | |
| 13 | | Natl Taiwan Univ | 8 | | | Aarhus Univ | | 0.04 | |
| 14 | | Wenzhou Med Univ | 7 | | | China Med Univ Hosp | | 0.04 | |
| 15 | | Harvard TH Chan Sch Publ Hlth | 7 | | | Zhejiang Univ | | 0.04 | |
| 16 | | Harvard Med Sch | 7 | | | Albert Einstein Coll Med | | 0.03 | |
| 17 | | Nanjing Med Univ | 6 | | | Harvard Univ | | 0.03 | |
| 18 | | Peking Univ | 6 | | | Natl Taiwan Univ | | 0.03 | |
| 19 | | Baylor Coll Med | 6 | | | Harvard TH Chan Sch Publ Hlth | | 0.03 | |
| 20 | | Univ South Carolina | 5 | | | Univ South Carolina | | 0.03 | |
| 21 | | Sun Yat Sen Univ | 5 | | | Capital Med Univ | | 0.03 | |
| 22 | | Univ London Imperial Coll Sci Technol & Med | 5 | | | Sungkyunkwan Univ | | 0.02 | |
| 23 | | CHA Univ | 5 | | | Karolinska Inst | | 0.02 | |
| 24 | | Canc Registry Norway | 5 | | | Columbia Univ | | 0.02 | |
| 25 | | Aarhus Univ | 5 | | | Univ Utah | | 0.02 | |
| 26 | | Capital Med Univ | 5 | | | Amer Canc Soc | | 0.02 | |
| 27 | | Cedars Sinai Med Ctr | 5 | | | Univ Oxford | | 0.02 | |
| 28 | | Chongqing Med Univ | 4 | | | Asia Univ | | 0.02 | |
| 29 | | Karolinska Inst | 4 | | | Univ Washington | | 0.02 | |
| 30 | | Shanghai Jiao Tong Univ | 4 | | | Univ Pittsburgh | | 0.02 | |
| 31 | | Univ Toronto | 4 | | | Chang Gung Univ | | 0.01 | |
| 32 | | China Med Univ | 4 | | | Peking Univ | | 0.01 | |
| 33 | | Columbia Univ | 4 | | | Univ London Imperial Coll Sci Technol & Med | | 0.01 | |
| 34 | | Univ Cambridge | 4 | | | CHA Univ | | 0.01 | |
| 35 | | Massachusetts Gen Hosp | 4 | | | Boston Univ | | 0.01 | |
| 36 | | Boston Univ | 4 | | | Univ Malaya | | 0.01 | |
| 37 | | Med Univ Vienna | 4 | | | Connecting Hlth Innovat LLC | | 0.01 | |
| 38 | | Natl Taiwan Univ Hosp | 4 | | | Chung Shan Med Univ | | 0.01 | |
| 39 | | Univ Verona | 4 | | | Weill Cornell Med Coll | | 0.01 | |
| 40 | | Chinese Univ Hong Kong | 4 | | | Canc Res & Prevent Inst ISPO | | 0.01 | |
| 41 | | Seoul Natl Univ Hosp | 4 | | | Chinese Peoples Liberat Army Gen Hosp | | 0.01 | |
| 42 | | Univ Malaya | 4 | | | Acad Athens | | 0.01 | |
| 43 | | Connecting Hlth Innovat LLC | 4 | | | Beth Israel Deaconess Med Ctr | | 0.01 | |
| 44 | | Duke Univ | 3 | | | Univ Alabama Birmingham | | 0.01 | |
| 45 | | Univ Utah | 3 | | | Stanford Univ | | 0.01 | |
| 46 | | China Med Univ Hosp | 3 | | | Univ Milan | | 0.01 | |
| 47 | | Saga Univ | 3 | | | Univ Calif Berkeley | | 0.01 | |
| 48 | | Chinese Acad Med Sci | 3 | | | Vanderbilt Univ | | 0.01 | |
| 49 | | Chung Shan Med Univ | 3 | | | Basque Reg Hlth Dept | | 0.01 | |
| 50 | | Cent South Univ | 3 | | | Chinese Acad Med Sci & Peking Union Med Coll | | 0.01 | |
| 51 | | Amer Canc Soc | 3 | | | Addenbrookes Hosp | | 0.01 | |
| 52 | | Chang Gung Mem Hosp | 3 | | | Univ Athens | | 0.01 | |
| 53 | | Kagawa Univ | 3 | | | Chung Ang Univ | | 0.01 | |
| 54 | | Yokohama City Univ Med | 3 | | | Fujian Med Univ | | 0 | |
| 55 | | Zhejiang Univ | 3 | | | China Japan Friendship Hosp | | 0 | |
| **４.２Top 10 institutions and associated countries** | | | | | | | | |  |
| Rank | Institution | | | Country | Institution | | Country | |  |
| 1 | Sungkyunkwan Univ | | | Korea | Dana Farber Canc Inst | | USA | |  |
| 2 | Seoul Natl Univ | | | Korea | German Canc Res Ctr | | Germany | |  |
| 3 | Yonsei Univ | | | Korea | Yonsei Univ | | Korea | |  |
| 4 | Fujian Med Univ | | | China | Nanjing Med Univ | | China | |  |
| 5 | Brigham & Womens Hosp | | | USA | China Med Univ | | China | |  |
| 6 | China Japan Friendship Hosp | | | China | Univ Cambridge | | UK | |  |
| 7 | Albert Einstein Coll Med | | | USA | Massachusetts Gen Hosp | | USA | |  |
| 8 | Harvard Univ | | | USA | Brigham & Womens Hosp | | USA | |  |
| 9 | Dana Farber Canc Inst | | | USA | Soochow Univ | | China | |  |
| 10 | Fujian Canc Hosp | | | China | Seoul Natl Univ | | Korea | |  |

# 5.Author

| **The authors ranked by the number of articles and centrality** | | | | |
| --- | --- | --- | --- | --- |
| **Ranking** | **Authors** | **Article** | **Authors** | **Centrality** |
| 1 | DONG IL PARK | 15 | THOMAS E ROHAN | 0.01 |
| 2 | YOON SUK JUNG | 14 | DONG IL PARK | 0 |
| 3 | FENG PENG | 14 | YOON SUK JUNG | 0 |
| 4 | DAN HU | 14 | FENG PENG | 0 |
| 5 | XIONGWEI ZHENG | 12 | DAN HU | 0 |
| 6 | HEJUN ZHANG | 12 | XIONGWEI ZHENG | 0 |
| 7 | WENQUAN NIU | 12 | HEJUN ZHANG | 0 |
| 8 | XIANDONG LIN | 11 | WENQUAN NIU | 0 |
| 9 | JINXIU LIN | 11 | XIANDONG LIN | 0 |
| 10 | CHONG IL SOHN | 10 | JINXIU LIN | 0 |
| 11 | JUNG HO PARK | 10 | CHONG IL SOHN | 0 |
| 12 | YAN XIA | 10 | JUNG HO PARK | 0 |
| 13 | NAM HEE KIM | 9 | YAN XIA | 0 |
| 14 | BINYING LIANG | 8 | NAM HEE KIM | 0 |
| 15 | ANDREW T CHAN | 7 | BINYING LIANG | 0 |
| 16 | JAMES R HEBERT | 7 | ANDREW T CHAN | 0 |
| 17 | NITIN SHIVAPPA | 7 | JAMES R HEBERT | 0 |
| 18 | THOMAS E ROHAN | 6 | NITIN SHIVAPPA | 0 |
| 19 | EDWARD L GIOVANNUCCI | 6 | EDWARD L GIOVANNUCCI | 0 |
| 20 | GANG CHEN | 6 | GANG CHEN | 0 |
| 21 | SUNG NOH HONG | 5 | SUNG NOH HONG | 0 |
| 22 | CHAO LI | 5 | CHAO LI | 0 |
| 23 | KYUYONG CHOI | 4 | KYUYONG CHOI | 0 |
| 24 | GUOHUI FAN | 4 | GUOHUI FAN | 0 |
| 25 | KYUNGDO HAN | 4 | KYUNGDO HAN | 0 |
| 26 | DAVID NIEDERSEER | 4 | DAVID NIEDERSEER | 0 |
| 27 | DONG KYUNG CHANG | 4 | DONG KYUNG CHANG | 0 |
| 28 | YOUNGHO KIM | 4 | YOUNGHO KIM | 0 |
| 29 | YING CHEN | 4 | YING CHEN | 0 |
| 30 | SEUNGHO RYU | 4 | SEUNGHO RYU | 0 |
| 31 | BERNHARD WERNLY | 4 | BERNHARD WERNLY | 0 |
| 32 | ELMAR AIGNER | 4 | ELMAR AIGNER | 0 |
| 33 | HEE JUNG SON | 4 | HEE JUNG SON | 0 |
| 34 | SARAH WERNLY | 4 | SARAH WERNLY | 0 |
| 35 | GEORG SEMMLER | 4 | GEORG SEMMLER | 0 |
| 36 | CHRISTIAN DATZ | 4 | CHRISTIAN DATZ | 0 |
| 37 | ABBY B SIEGEL | 3 | ABBY B SIEGEL | 0 |
| 38 | HIDEO YASUNAGA | 3 | HIDEO YASUNAGA | 0 |
| 39 | TATSUYA KAMON | 3 | TATSUYA KAMON | 0 |
| 40 | AKIRA NISHIYAMA | 3 | AKIRA NISHIYAMA | 0 |
| 41 | HIROYUKI MORITA | 3 | HIROYUKI MORITA | 0 |
| 42 | AMANDA J CROSS | 3 | AMANDA J CROSS | 0 |
| 43 | KOJIRO MORITA | 3 | KOJIRO MORITA | 0 |
| 44 | YUICHIRO YANO | 3 | YUICHIRO YANO | 0 |
| 45 | KOICHI NODE | 3 | KOICHI NODE | 0 |
| 46 | HIROYUKI KIRIYAMA | 3 | HIROYUKI KIRIYAMA | 0 |
| 47 | NOBUAKI MICHIHATA | 3 | NOBUAKI MICHIHATA | 0 |
| 48 | TAISUKE JO | 3 | TAISUKE JO | 0 |
| 49 | GUODONG XU | 3 | GUODONG XU | 0 |
| 50 | HIDETAKA ITOH | 3 | HIDETAKA ITOH | 0 |
| 51 | KATSUHITO FUJIU | 3 | KATSUHITO FUJIU | 0 |
| 52 | HIDEHIRO KANEKO | 3 | HIDEHIRO KANEKO | 0 |
| 53 | ADEYINKA O LAIYEMO | 3 | ADEYINKA O LAIYEMO | 0 |
| 54 | ISSEI KOMURO | 3 | ISSEI KOMURO | 0 |
| 55 | NORIFUMI TAKEDA | 3 | NORIFUMI TAKEDA | 0 |

# 6.Co-Citation information

| **The top 40 highly Co-cited referances from VOSviewer** | | |
| --- | --- | --- |
| **Rank** | **Label** | **Citations** |
| 1 | ahmed rl, 2006, cancer-am cancer soc, v107, p28, doi 10.1002/cncr.21950 | 75 |
| 2 | giovannucci e, 2007, am j clin nutr, v86, p836s, doi 10.1093/ajcn/86.3.836s | 65 |
| 3 | esposito k, 2012, diabetes care, v35, p2402, doi 10.2337/dc12-0336 | 55 |
| 4 | esposito k, 2013, endocrine, v44, p634, doi 10.1007/s12020-013-9939-5 | 46 |
| 5 | kim jh, 2007, cancer epidem biomar, v16, p1543, doi 10.1158/1055-9965.epi-07-0199 | 44 |
| 6 | bardou m, 2013, gut, v62, p933, doi 10.1136/gutjnl-2013-304701 | 41 |
| 7 | renehan ag, 2008, lancet, v371, p569, doi 10.1016/s0140-6736(08)60269-x | 39 |
| 8 | stocks t, 2011, cancer-am cancer soc, v117, p2398, doi 10.1002/cncr.25772 | 37 |
| 9 | cowey s, 2006, am j pathol, v169, p1505, doi 10.2353/ajpath.2006.051090 | 36 |
| 10 | aleksandrova k, 2011, cancer prev res, v4, p1873, doi 10.1158/1940-6207.capr-11-0218 | 35 |
| 11 | calle ee, 2003, new engl j med, v348, p1625, doi 10.1056/nejmoa021423 | 35 |
| 12 | jinjuvadia r, 2013, j clin gastroenterol, v47, p33, doi 10.1097/mcg.0b013e3182688c15 | 35 |
| 13 | liu cs, 2010, bmc gastroenterol, v10, doi 10.1186/1471-230x-10-51 | 34 |
| 14 | alberti kgmm, 2009, circulation, v120, p1640, doi 10.1161/circulationaha.109.192644 | 33 |
| 15 | lieberman da, 2012, gastroenterology, v143, p844, doi 10.1053/j.gastro.2012.06.001 | 31 |
| 16 | stocks t, 2008, int j obesity, v32, p304, doi 10.1038/sj.ijo.0803713 | 31 |
| 17 | wong vws, 2011, gut, v60, p829, doi 10.1136/gut.2011.237974 | 29 |
| 18 | colangelo la, 2002, cancer epidem biomar, v11, p385 | 28 |
| 19 | kim bc, 2012, cancer cause control, v23, p727, doi 10.1007/s10552-012-9942-9 | 28 |
| 20 | pais r, 2009, world j gastroentero, v15, p5141, doi 10.3748/wjg.15.5141 | 27 |
| 21 | bray f, 2018, ca-cancer j clin, v68, p394, doi 10.3322/caac.21492 | 26 |
| 22 | grundy sm, 2005, circulation, v112, p2735, doi 10.1161/circulationaha.105.169404 | 26 |
| 23 | larsson sc, 2007, am j clin nutr, v86, p556, doi 10.1093/ajcn/86.3.556 | 25 |
| 24 | cleeman ji, 2001, jama-j am med assoc, v285, p2486, doi 10.1001/jama.285.19.2486 | 24 |
| 25 | giovannucci e, 2001, j nutr, v131, p3109s, doi 10.1093/jn/131.11.3109s | 24 |
| 26 | larsson sc, 2005, jnci-j natl cancer i, v97, p1679, doi 10.1093/jnci/dji375 | 24 |
| 27 | morita takako, 2005, asian pac j cancer prev, v6, p485,PMID: 16435997 | 23 |
| 28 | ben qw, 2012, gastroenterology, v142, p762, doi 10.1053/j.gastro.2011.12.050 | 22 |
| 29 | hwang st, 2010, j gastroen hepatol, v25, p562, doi 10.1111/j.1440-1746.2009.06117.x | 22 |
| 30 | jemal a, 2011, ca-cancer j clin, v61, p69, doi 10.3322/caac.20107 | 22 |
| 31 | otake s, 2005, clin cancer res, v11, p3642, doi 10.1158/1078-0432.ccr-04-1868 | 22 |
| 32 | stadlmayr a, 2011, j intern med, v270, p41, doi 10.1111/j.1365-2796.2011.02377.x | 22 |
| 33 | trevisan m, 2001, cancer epidem biomar, v10, p937 | 22 |
| 34 | ferlay j, 2015, int j cancer, v136, pe359, doi 10.1002/ijc.29210 | 21 |
| 35 | okabayashi k, 2012, am j gastroenterol, v107, p1175, doi 10.1038/ajg.2012.180 | 21 |
| 36 | chiu hm, 2007, clin gastroenterol h, v5, p221, doi 10.1016/j.cgh.2006.06.022 | 20 |
| 37 | kang hw, 2010, am j gastroenterol, v105, p178, doi 10.1038/ajg.2009.541 | 20 |
| 38 | ma yl, 2013, plos one, v8, doi 10.1371/journal.pone.0053916 | 20 |
| 39 | pelucchi c, 2010, eur j cancer, v46, p1866, doi 10.1016/j.ejca.2010.03.010 | 20 |
| 40 | pischon t, 2006, j natl cancer i, v98, p920, doi 10.1093/jnci/djj246 | 20 |

# 7. References

**The top 10 highly cited references and co-cited references.**

| Items | Rank | Title | Citations | Years |
| --- | --- | --- | --- | --- |
| Cited references | 1 | The gut microbiota and host health: a new clinical frontier | 1349 | 2016 |
|  | 2 | Obesity and colorectal cancer | 486 | 2013 |
|  | 3 | The role of the microbiome in cancer development and therapy | 339 | 2017 |
|  | 4 | Intestinal microbiota in health and disease: Role of bifidobacteria in gut homeostasis | 300 | 2014 |
|  | 5 | Ellagic Acid Metabolism by Human Gut Microbiota: Consistent Observation of Three Urolithin Phenotypes in Intervention Trials, Independent of Food Source, Age, and Health Status | 249 | 2014 |
|  | 6 | Complications, morbidity and mortality of nonalcoholic fatty liver disease | 238 | 2020 |
|  | 7 | Links of gut microbiota composition with alcohol dependence syndrome and alcoholic liver disease | 225 | 2017 |
|  | 8 | Obesity and cancer: inflammation bridges the two | 214 | 2016 |
|  | 9 | Tumor-Induced IL-6 Reprograms Host Metabolism to Suppress Anti-tumor Immunity | 213 | 2016 |
|  | 10 | Hypomagnesemia is a significant predictor of cardiovascular and non-cardiovascular mortality in patients undergoing hemodialysis | 211 | 2014 |
| Co-cited references | 1 | The Metabolic Syndrome and Risk of Incident Colorectal Cancer | 75 | 2006 |
|  | 2 | Metabolic syndrome, hyperinsulinemia, and colon cancer: a review | 65 | 2007 |
|  | 3 | Metabolic syndrome and risk of cancer: a systematic review and meta-analysis | 55 | 2012 |
|  | 4 | Colorectal cancer association with metabolic syndrome and its components: a systematic review with meta-analysis | 46 | 2013 |
|  | 5 | Is metabolic syndrome a risk factor for colorectal adenoma? | 44 | 2007 |
|  | 6 | Obesity and colorectal cancer | 41 | 2013 |
|  | 7 | Body-mass index and incidence of cancer: a systematic review and meta-analysis of prospective observational studies | 39 | 2008 |
|  | 8 | Metabolic factors and the risk of colorectal cancer in 580,000 men and women in the metabolic syndrome and cancer project (Me-Can) | 37 | 2011 |
|  | 9 | The metabolic syndrome: A high-risk state for cancer? | 36 | 2006 |
|  | 10 | Metabolic syndrome and risks of colon and rectal cancer: the European prospective investigation into cancer and nutrition study | 35 | 2011 |

# 8.Citation Bursts

| **Top 20 References with the Strongest Citation Bursts** | | |
| --- | --- | --- |
| **Burst year** | **DOI** | **Title** |
| 2013-2015 | 10.1186/1471-230X-10-51 | Central obesity and atherogenic dyslipidemia in metabolic syndrome are associated with increased risk for colorectal adenoma in a Chinese population |
|  | 10.1002/ijc.25516 | Estimates of worldwide burden of cancer in 2008: GLOBOCAN 2008 |
|  | 10.1097/MAJ.0b013e3181df9055 | Metabolic Syndrome and Its Association With Colorectal Cancer: A Review |
|  | 10.1002/cncr.25772 | Metabolic factors and the risk of colorectal cancer in 580,000 men and women in the metabolic syndrome and cancer project (Me-Can) |
|  | 10.1111/j.1349-7006.2010.01518.x | Adipocytokines as new promising markers of colorectal tumors: Adiponectin for colorectal adenoma, and resistin and visfatin for colorectal cancer |
|  | 10.2337/dc09-1197 | Visceral Fat Area and Markers of Insulin Resistance in Relation to Colorectal Neoplasia |
|  | 10.1186/1471-2350-12-94 | Effects of genetic variations in the Adiponectin pathway genes on the risk of colorectal cancer in the Chinese population |
| 2013-2016 | 10.1158/1940-6207.CAPR-11-0218 | Metabolic Syndrome and Risks of Colon and Rectal Cancer: The European Prospective Investigation into Cancer and Nutrition Study |
| 2014-2017 | 10.2337/dc12-0336 | Metabolic Syndrome and Risk of Cancer: A systematic review and meta-analysis |
| 2015-2017 | 10.1053/j.gastro.2012.06.001 | Guidelines for Colonoscopy Surveillance After Screening and Polypectomy: A Consensus Update by the US Multi-Society Task Force on Colorectal Cancer |
|  | 10.1038/ajg.2012.180 | Body Mass Index Category as a Risk Factor for Colorectal Adenomas: A Systematic Review and Meta-Analysis |
|  | 10.1136/gutjnl-2013-304701 | Obesity and colorectal cancer |
| 2015-2018 | 10.1007/s12020-013-9939-5 | Colorectal cancer association with metabolic syndrome and its components: a systematic review with meta-analysis |
| 2016-2019 | 10.1016/j.cgh.2014.10.022 | Effects of Metabolic Syndrome and Findings From Baseline Colonoscopies on Occurrence of Colorectal Neoplasms |
| 2016-2020 | 10.1002/ijc.29210 | Cancer incidence and mortality worldwide: Sources, methods and major patterns in GLOBOCAN 2012 |
| 2017-2019 | 10.1002/ijc.30404 | Preoperative metabolic syndrome and prognosis after radical resection for colorectal cancer: The Fujian prospective investigation of cancer (FIESTA) study |
|  | 10.3322/caac.21338 | Cancer statistics in China, 2015 |
| 2017-2020 | 10.1016/j.metabol.2014.10.008 | Metabolic syndrome and risk of cancer: Which link? |
| 2018-2022 | 10.1136/gutjnl-2015-310912 | Global patterns and trends in colorectal cancer incidence and mortality |
| 2019-2022 | 10.1007/s10654-018-0440-6 | Abdominal obesity, glucose intolerance and decreased high-density lipoprotein cholesterol as components of the metabolic syndrome are associated with the development of colorectal cancer |

# 9.Keywords Cluster

| **Keywords cluster analysis (the silhouette value is over 0.7)** | | | | | |
| --- | --- | --- | --- | --- | --- |
| **Ccluster** | **Size** | **Sihouette** | **Mean Years** | **Label（LLR）** | **Other Keywords** |
| 0 | 43 | 0.859 | 2015 | adiponectin | adiponectin (31.15, 1.0E-4); leptin (13.42, 0.001); chemerin (7.41, 0.01); igf-1 (6.72, 0.01); cytokines (5.81, 0.05); carcinoembryonic antigen (5.81, 0.05); polymorphism (5.81, 0.05); electronic nose (5.54, 0.05); metabolic parameter (5.54, 0.05); nurses health (5.54, 0.05); snoring (5.54, 0.05); in vitro (5.54, 0.05); pai 1 (5.54, 0.05); cyclooxygenase-2 (5.54, 0.05); beige adipocyte (5.54, 0.05); system (5.54, 0.05); erk (5.54, 0.05); gas sensors (5.54, 0.05); molecular weight adiponectin (5.54, 0.05); serum lipids (5.54, 0.05); metabolic factor (5.54, 0.05); hdac2 mrna (5.54, 0.05); 12 dimethylhydrazine (dmh) (5.54, 0.05); biological age (5.54, 0.05); growth factor (5.54, 0.05); volatile organic compounds (5.54, 0.05); streptozotocin (stz) (5.54, 0.05); colon health (5.54, 0.05); cancer antigens (5.54, 0.05); esophageal adenocarcinoma (5.54, 0.05); cancer cachexia (5.54, 0.05); clonogenic assay (5.54, 0.05); single-nucleotide polymorphism (5.54, 0.05); time-restricted feeding (trf) (5.54, 0.05); ki-67 (5.54, 0.05); myocardial infarction (5.54, 0.05); serum adiponectin (5.54, 0.05); chronological age (5.54, 0.05); scratch wound healing assay (5.54, 0.05); diagnostic marker (5.54, 0.05); age gap (5.54, 0.05); lifestyle factors (5.54, 0.05); serum lipid (5.54, 0.05); fto (5.54, 0.05); northern sweden (5.54, 0.05); receptor (5.54, 0.05); lipolysis (5.54, 0.05); clinical trials (5.54, 0.05); adipor1 (5.54, 0.05); postmenopausal (5.54, 0.05); physical activity questionnaire (5.54, 0.05); urine (5.54, 0.05); leukocyte count (5.54, 0.05); emulsifiers (5.54, 0.05); adiponectin receptor (5.54, 0.05); barretts esophagus (5.54, 0.05); quicki (5.54, 0.05); adipokine (5.54, 0.05); inflammatory bowel disease (ibd) (5.54, 0.05); glut4 (5.54, 0.05); subclinical inflammation (5.54, 0.05); tumor necrosis factor alpha (5.54, 0.05); heart disease (5.54, 0.05); kimchi (5.54, 0.05); plasma leptin (5.54, 0.05); resistin (4.75, 0.05); mortality (4.12, 0.05); diabetes mellitus (3.99, 0.05); prostate cancer (3.97, 0.05); colorectal neoplasms (3.86, 0.05); postmenopausal women (3.36, 0.1); adipocytokine (3.36, 0.1); biomarkers (3.36, 0.1); inflammation (3.1, 0.1); colonoscopy (3.08, 0.1); survival (3.08, 0.1); fitness (2.9, 0.1); linoleic acid (2.9, 0.1); cancer biology (2.9, 0.1); tnf-alpha (2.9, 0.1); african americans (2.9, 0.1); glucose intolerance (2.9, 0.1); ghrelin (2.9, 0.1); weight gain (2.9, 0.1); visceral obesity (2.9, 0.1); anxiety (2.9, 0.1); colorectal malignant neoplasm (2.9, 0.1); synchronizer (2.9, 0.1); skin tag (2.9, 0.1); prognostic factor (2.9, 0.1); homa-ir (2.9, 0.1); adipocytokines (2.9, 0.1); mendelian randomization (2.9, 0.1); hydroxyoctadecadienoic acids (2.9, 0.1); visfatin (2.9, 0.1); insulin (2.61, 0.5); hepatocellular carcinoma (2.46, 0.5); breast cancer (2.36, 0.5); diabetes (2.3, 0.5); diet (2.3, 0.5) |
| 1 | 33 | 0.828 | 2015 | gene expression | gene expression (12.05, 0.001); visceral fat (8.34, 0.005); adipose tissue (8.02, 0.005); advanced adenoma (6.71, 0.01); chemotherapy (6.71, 0.01); abdominal computed tomography (6.02, 0.05); activation (6.02, 0.05); neopterin (6.02, 0.05); lipopolysaccharide binding protein (6.02, 0.05); multi-omics (6.02, 0.05); outcome (6.02, 0.05); clinicopathological feature (6.02, 0.05); hhex (6.02, 0.05); single nucleotide polymorphism (6.02, 0.05); abdominal fat (6.02, 0.05); genetic variants (6.02, 0.05); beta-catenin (6.02, 0.05); transcriptome (6.02, 0.05); serum concentration (6.02, 0.05); ppar gamma agonists (6.02, 0.05); drug sensitivity (6.02, 0.05); colorectal cancer risk (6.02, 0.05); radiation therapy (6.02, 0.05); contribute (6.02, 0.05); intestinal permeability (6.02, 0.05); hepatocyte growth factor (6.02, 0.05); metabolic status (6.02, 0.05); genome-wide association study (6.02, 0.05); barrier function (6.02, 0.05); rectum surgery (6.02, 0.05); cancer death (6.02, 0.05); cell-mediated immunity (6.02, 0.05); waist-to-hip ratio (6.02, 0.05); racial disparities (6.02, 0.05); pleiotropy (6.02, 0.05); proliferation (4.82, 0.05); inflammation (3.43, 0.1); cladosporols (3.35, 0.1); machine learning (3.35, 0.1); blood glucose (3.35, 0.1); alpha-fetoprotein (3.35, 0.1); metabolic risk factors (3.35, 0.1); trend estimation (3.35, 0.1); african americans (3.35, 0.1); cell cycle arrest (3.35, 0.1); artificial intelligence (3.35, 0.1); adipocytes (3.35, 0.1); us adult (3.35, 0.1); adipogenesis (3.35, 0.1); bmi (3.35, 0.1); liver steatosis (3.35, 0.1); korean men (3.35, 0.1); hyperglycemia (3.35, 0.1); treatment related toxicity (3.35, 0.1); prognostic factor (3.35, 0.1); homa-ir (3.35, 0.1); obese-related adipokines (3.35, 0.1); migration (3.35, 0.1); metabolic syndrome (mets) (3.35, 0.1); phytochemicals (3.35, 0.1); adipokines (3.35, 0.1); visfatin (3.35, 0.1); epidemiology (2.51, 0.5); cancer (2.42, 0.5); colorectal cancer screening (2.4, 0.5); fat accumulation (2.4, 0.5); colorectal cancer (crc) (2.4, 0.5); type 2 diabetes mellitus (2.4, 0.5); outcm (2.4, 0.5); risk factor (2.2, 0.5); component (1.83, 0.5); surveillance (1.83, 0.5); chemotherapy resistance (1.83, 0.5); caloric restriction (1.83, 0.5); glargine (1.83, 0.5); complications (1.83, 0.5); disease (1.83, 0.5); elevated glucose (1.83, 0.5); impact (1.83, 0.5); visceral adipose tissue (1.83, 0.5); glycosylated hemoglobin (1.83, 0.5); computed tomography (1.83, 0.5); diet (1.8, 0.5); breast cancer (1.8, 0.5); colon (1.49, 0.5); colorectal neoplasm (1.49, 0.5); endometrial cancer (1.42, 0.5); resistin (1.42, 0.5); waist circumference (1.42, 0.5); biomarker (1.42, 0.5); cohort study (1.39, 0.5); risk (1.39, 0.5); meta-analysis (1.29, 0.5); insulin (1.25, 0.5); diabetes mellitus (1.19, 0.5); dietary inflammatory index (1.19, 0.5); physical activity (1.19, 0.5); colorectal adenoma (1.16, 0.5); apoptosis (1.12, 0.5); chemoprevention (1.12, 0.5) |
| 2 | 33 | 0.911 | 2018 | metabolism | metabolism (14.85, 0.001); microbiome (14.85, 0.001); cachexia (14.29, 0.001); microbiota (11.16, 0.001); next generation sequencing (7.13, 0.01); faecal microbiota transplantation (7.13, 0.01); msh2 (7.13, 0.01); localization (7.13, 0.01); protein– (7.13, 0.01); ras paralogs (7.13, 0.01); cancers (7.13, 0.01); miscarriages (7.13, 0.01); akt (7.13, 0.01); interactome (7.13, 0.01); susceptibility (7.13, 0.01); ngs (7.13, 0.01); absorption (7.13, 0.01); dna mismatch repair proteins (7.13, 0.01); bilirubin (7.13, 0.01); 5-mthf (7.13, 0.01); epcam (7.13, 0.01); gametes (7.13, 0.01); folic acid (7.13, 0.01); unconjugated bilirubin (7.13, 0.01); sugar (7.13, 0.01); mycobiota (7.13, 0.01); immunotherapy (7.13, 0.01); embryos (7.13, 0.01); enos (7.13, 0.01); mitochondrial dysfunction (7.13, 0.01); overexpression (7.13, 0.01); aerobic glycolysis (7.13, 0.01); umfa (7.13, 0.01); cytochrome c oxidase (7.13, 0.01); host (7.13, 0.01); neutrophils (7.13, 0.01); bioid (7.13, 0.01); 16s rrna gene (7.13, 0.01); mtor (7.13, 0.01); mthfr (7.13, 0.01); ampk (7.13, 0.01); nitric oxide (7.13, 0.01); caenorhabditis elegans (7.13, 0.01); protein interaction (7.13, 0.01); sco2 (7.13, 0.01); causal inference (7.13, 0.01); glucocorticoid hormones (4.42, 0.05); cell (4.42, 0.05); genetic susceptibility (4.42, 0.05); methylglyoxal (4.42, 0.05); lipid droplets (4.42, 0.05); carbonyl stress (4.42, 0.05); mendelian randomization (4.42, 0.05); anti-interleukin-6 receptor antibody (4.42, 0.05); bacteria (3.43, 0.1); shift (3.43, 0.1); gastrointestinal diseases (3.43, 0.1); chain fatty acid (3.43, 0.1); intestinal microbiome (3.43, 0.1); fermentation (2.81, 0.1); fruit (2.81, 0.1); visceral adipose tissue (2.81, 0.1); polymorphism (2.81, 0.1); united states (2.81, 0.1); colorectal adenoma (2.65, 0.5); inflammatory bowel disease (2.36, 0.5); obesity (2.1, 0.5); expression (2.01, 0.5); mortality (1.83, 0.5); diabetes mellitus (1.78, 0.5); health (1.73, 0.5); colorectal neoplasms (1.72, 0.5); body mass index (1.66, 0.5); metabolic syndrome (1.54, 0.5); oxidative stress (1.5, 0.5); prognosis (1.43, 0.5); epidemiology (1.43, 0.5); colonoscopy (1.37, 0.5); survival (1.37, 0.5); adiponectin (1.31, 0.5); nafld (1.31, 0.5); risk factor (1.25, 0.5); diabetes (1.02, 0.5); breast cancer (1.02, 0.5); hypertension (1.02, 0.5); association (1.02, 0.5); insulin (0.96, 0.5); recurrence (0.91, 0.5); risk factors (0.91, 0.5); inflammation (0.89, 0.5); colorectal neoplasm (0.85, 0.5); cohort study (0.79, 0.5); risk (0.79, 0.5); sarcopenia (0.73, 0.5); meta-analysis (0.73, 0.5); screening (0.67, 0.5); dietary inflammatory index (0.67, 0.5); physical activity (0.67, 0.5); dyslipidemia (0.62, 0.5); leptin (0.62, 0.5) |
| 3 | 32 | 0.857 | 2016 | diabetes | diabetes (16.15, 1.0E-4); colonoscopy (15.86, 1.0E-4); colorectal polyps (15.82, 1.0E-4); colorectal neoplasms (12.38, 0.001); colorectal neoplasm (9.86, 0.005); recurrence (9.21, 0.005); adenocarcinoma (7.35, 0.01); colorectal polyp (7.35, 0.01); pre-diabetes (7.35, 0.01); surveillance (5.76, 0.05); triglycerides (5.76, 0.05); health examination (5.51, 0.05); gallbladder (5.51, 0.05); preoperative period (5.51, 0.05); risk stratifying tool (5.51, 0.05); teeth disease (5.51, 0.05); risk assessment (5.51, 0.05); helicobacter pylori infection (5.51, 0.05); immune system (5.51, 0.05); turkey (5.51, 0.05); precancerous lesions (5.51, 0.05); mass screening (5.51, 0.05); tumour location (5.51, 0.05); eggers regression test (5.51, 0.05); sglt2 inhibitor (5.51, 0.05); random forest (5.51, 0.05); predictive model (5.51, 0.05); type 2 diabetic mellitus (5.51, 0.05); allergic disease (5.51, 0.05); nomogram (5.51, 0.05); anatomical location (5.51, 0.05); colonscopy (5.51, 0.05); metabolic syndrome x (5.51, 0.05); low bone mineral density (5.51, 0.05); renal cancer (5.51, 0.05); young adult (5.51, 0.05); non-invasive (5.51, 0.05); trims and fill analysis (5.51, 0.05); death (5.51, 0.05); lipids (4.7, 0.05); mortality (4.18, 0.05); diabetes mellitus (4.05, 0.05); cancer (3.95, 0.05); triglyceride (3.92, 0.05); body mass index (3.79, 0.1); epidemiology (3.26, 0.1); adiponectin (2.99, 0.1); gastric polyps (2.87, 0.1); hba(1c) (2.87, 0.1); non-neoplastic polyps (2.87, 0.1); non-small cell lung cancer (2.87, 0.1); health check (2.87, 0.1); personalized platelet count (2.87, 0.1); society task force (2.87, 0.1); advanced adenomatous polyps (2.87, 0.1); tumor (2.87, 0.1); research (2.87, 0.1); gallbladder polyps (2.87, 0.1); advanced neoplasia (2.87, 0.1); liver disease (2.87, 0.1); pathology (2.87, 0.1); rectal neoplasms (2.87, 0.1); postoperative period (2.87, 0.1); fatty liver index (2.87, 0.1); insulin-like growth factor (2.87, 0.1); regression (2.87, 0.1); hemoglobin-to-platelet ratio (2.87, 0.1); occult blood (2.87, 0.1); hyperglycaemia (2.87, 0.1); young adults (2.87, 0.1); cirrhosis (2.87, 0.1); gastroscopy (2.87, 0.1); gleason score (2.87, 0.1); cardiovascular (2.87, 0.1); korea (2.87, 0.1); cohort of norway (2.87, 0.1); metabolic syndrome (mets) (2.87, 0.1); beta-catenin accumulated crypts (2.87, 0.1); metabolic risk score (2.87, 0.1); lymphocyte-to-monocyte ratio (2.87, 0.1); rectum (2.87, 0.1); non-advanced adenomatous polyps (2.87, 0.1); non-alcoholic fatty liver disease (2.82, 0.1); insulin resistance (2.64, 0.5); adenoma (2.42, 0.5); diet (2.33, 0.5); breast cancer (2.33, 0.5); hypertension (2.33, 0.5); insulin (2.2, 0.5); inflammatory bowel diseases (1.96, 0.5); guideline (1.96, 0.5); helicobacter pylori (1.96, 0.5); nonalcoholic fatty liver (1.96, 0.5); liver (1.96, 0.5); cancer screening (1.96, 0.5); dyslipidaemia (1.96, 0.5); kras (1.96, 0.5); gastric neoplasm (1.96, 0.5); neutrophil-to-lymphocyte ratio (1.96, 0.5); intestinal microbiota (1.96, 0.5) |
| 4 | 32 | 0.928 | 2014 | abdominal obesity | abdominal obesity (16.06, 1.0E-4); transient elastography (6.81, 0.01); exercise treadmill test (6.81, 0.01); coronary artery disease (6.81, 0.01); gallbladder polyp (6.81, 0.01); hypokalemia (6.81, 0.01); ectopic fat accumulation (6.81, 0.01); hyponatremia (6.81, 0.01); acute renal failure (6.81, 0.01); consensus (6.81, 0.01); 25-hydroxyvitamin d levels (6.81, 0.01); heart rate recovery (6.81, 0.01); barretts oesophagus (6.81, 0.01); asia (6.81, 0.01); colorectal tissue (6.81, 0.01); young-adult (6.81, 0.01); oesophageal cancer (6.81, 0.01); perilipin (6.81, 0.01); postmenopausal hormone therapy (6.81, 0.01); villous adenoma (6.81, 0.01); body fat distribution (6.81, 0.01); metabolic acidosis (6.81, 0.01); cancer prevention (5.6, 0.05); hba(1c) (4.11, 0.05); metabolic abnormality (4.11, 0.05); dietary carbohydrates (4.11, 0.05); advanced colorectal neoplasm (4.11, 0.05); research (4.11, 0.05); glucose intolerance (4.11, 0.05); adult (4.11, 0.05); colorectal tumor (4.11, 0.05); recommendation (4.11, 0.05); antihypertensive agents (4.11, 0.05); hyperglycaemia (4.11, 0.05); lipid droplets (4.11, 0.05); body fat percentage (4.11, 0.05); vitamin d deficiency (4.11, 0.05); metabolic diseases (4.11, 0.05); inflammatory bowel diseases (3.13, 0.1); fatty liver (3.13, 0.1); glycemic load (3.13, 0.1); appetite (3.13, 0.1); liver (3.13, 0.1); gerd (3.13, 0.1); glycemic index (3.13, 0.1); gastrointestinal (3.13, 0.1); high-density lipoprotein cholesterol (3.13, 0.1); coronary artery calcification (3.13, 0.1); sleep duration (3.13, 0.1); diarrhea (3.13, 0.1); pre-diabetes (3.13, 0.1); colorectal neoplasms (2.77, 0.1); risk factors (2.52, 0.5); surgery (2.51, 0.5); screening colonoscopy (2.51, 0.5); sex difference (2.51, 0.5); liver fibrosis (2.51, 0.5); colorectal carcinoma (2.51, 0.5); visceral adipose tissue (2.51, 0.5); glycosylated hemoglobin (2.51, 0.5); inflammation (2.3, 0.5); mortality (2.16, 0.5); diabetes mellitus (2.09, 0.5); nash (2.08, 0.5); waist circumference (2.08, 0.5); colorectal adenomas (2.08, 0.5); body mass index (1.95, 0.5); age (1.74, 0.5); chemoprevention (1.74, 0.5); prognosis (1.68, 0.5); survival (1.61, 0.5); adiponectin (1.54, 0.5); risk factor (1.47, 0.5); atherosclerosis (1.47, 0.5); health (1.47, 0.5); cancer risk (1.47, 0.5); colonoscopy (1.34, 0.5); colorectal neoplasia (1.25, 0.5); microbiota (1.25, 0.5); epidemiology (1.24, 0.5); diet (1.2, 0.5); breast cancer (1.2, 0.5); hypertension (1.2, 0.5); association (1.2, 0.5); colorectal adenoma (1.16, 0.5); insulin (1.13, 0.5); colon (1, 0.5); risk (0.93, 0.5); sarcopenia (0.86, 0.5); dietary inflammatory index (0.79, 0.5); physical activity (0.79, 0.5); dyslipidemia (0.73, 0.5); leptin (0.73, 0.5); hyperinsulinemia (0.73, 0.5); screening (0.67, 0.5); metformin (0.59, 0.5); gut microbiota (0.59, 0.5); hepatocellular carcinoma (0.59, 0.5); prevalence (0.59, 0.5); the fiesta study (0.59, 0.5) |
| 5 | 31 | 0.952 | 2015 | body mass index | body mass index (18.27, 1.0E-4); colon cancer (16.83, 1.0E-4); component (9.12, 0.005); crc (8.92, 0.005); adiponectin level (8.92, 0.005); growth factor i (8.92, 0.005); colonic neoplasms (5.75, 0.05); ulcerative coliti (5.33, 0.05); pollution (4.46, 0.05); protective (4.46, 0.05); cause-effect (4.46, 0.05); vegetarian diet (4.46, 0.05); metabolite (4.46, 0.05); leptin concentration (4.46, 0.05); gene-gene interaction (4.46, 0.05); cancer-caused life loss (4.46, 0.05); statin use (4.46, 0.05); insulin-like growth factor 1 receptor (4.46, 0.05); traditional chinese medicine syndrome (4.46, 0.05); yamc (4.46, 0.05); crohns disease (4.46, 0.05); curcumin (4.46, 0.05); eradication (4.46, 0.05); mucosa associated microbiota (4.46, 0.05); age-standardized mortality (4.46, 0.05); cancer incidence pattern (4.46, 0.05); colonic neoplasm (4.46, 0.05); fecal microbiota (4.46, 0.05); dha (4.46, 0.05); short term outcm (4.46, 0.05); polyp (4.46, 0.05); apnsnps (4.46, 0.05); surgical outcomes (4.46, 0.05); stroke (4.46, 0.05); cdh13 (4.46, 0.05); colon polyp (4.46, 0.05); visceral fat accumulation (4.46, 0.05); human colonic microbiota (4.46, 0.05); mets (4.46, 0.05); rs3865188 (4.46, 0.05); mc38 (4.46, 0.05); gas chromatography-mass spectrometry (4.46, 0.05); escherichia coli (4.46, 0.05); laparoscopic surgery (4.46, 0.05); fatty liver disease (4.46, 0.05); molecular mechanism (4.46, 0.05); postoperative (4.46, 0.05); physical activity (4.4, 0.05); breast cancer (4.05, 0.05); colorectal carcinoma (3.83, 0.1); cytokines (3.83, 0.1); impact (3.83, 0.1); insulin receptor (3.83, 0.1); glycosylated hemoglobin (3.83, 0.1); diabetes mellitus (3.59, 0.1); resistin (2.87, 0.1); dietary inflammatory index (2.67, 0.5); obesity (2.23, 0.5); apoptosis (2.19, 0.5); colorectal polyps (1.98, 0.5); nafld (1.98, 0.5); cohort studies (1.98, 0.5); adenoma (1.98, 0.5); blood pressure (1.98, 0.5); c-reactive protein (1.98, 0.5); outcomes (1.91, 0.5); tyg index (1.91, 0.5); human subjects (1.91, 0.5); plasma (1.91, 0.5); systemic inflammatory response (1.91, 0.5); bmi change (1.91, 0.5); ppar ligand (1.91, 0.5); environment (1.91, 0.5); stem cells (1.91, 0.5); blood glucose (1.91, 0.5); nuclear receptors (1.91, 0.5); colorectal neoplasms metabolism (1.91, 0.5); chlorogenic acids (1.91, 0.5); cell signaling (1.91, 0.5); aspirin use (1.91, 0.5); trend estimation (1.91, 0.5); national surgical quality improvement program (1.91, 0.5); febrile episodes (1.91, 0.5); antipyretics (1.91, 0.5); food-derived bioactive compounds (1.91, 0.5); colorectal surgery (1.91, 0.5); 2 deoxy d glucose (1.91, 0.5); targeting survivin (1.91, 0.5); ghrelin (1.91, 0.5); gender role (1.91, 0.5); rectal neoplasms (1.91, 0.5); youden index (1.91, 0.5); bowel disease (1.91, 0.5); precancerous polyps (1.91, 0.5); methylglyoxal (1.91, 0.5); buddhist (1.91, 0.5); waist circumference change (1.91, 0.5); visceral obesity (1.91, 0.5); us adult (1.91, 0.5); glycolysis (1.91, 0.5) |
| 6 | 30 | 0.874 | 2017 | epidemiology | epidemiology (17.35, 1.0E-4); cohort study (16.83, 1.0E-4); women (7.94, 0.005); glycemic load (7.94, 0.005); quality of life (7.94, 0.005); glycemic index (7.94, 0.005); tea (5.82, 0.05); european prospective investigation into cancer and nutrition (5.82, 0.05); squamous cell cancer (5.82, 0.05); chronic disease (5.82, 0.05); metabolic equivalent of task (5.82, 0.05); endothelial function (5.82, 0.05); sugar containing beverages (5.82, 0.05); 24-h dietary recall (5.82, 0.05); coffee (5.82, 0.05); vitamin d supplementation (5.82, 0.05); single‐ (5.82, 0.05); comorbidity (5.82, 0.05); alcohol intake (5.82, 0.05); vitamin d status (5.82, 0.05); dry beans (5.82, 0.05); chronic disease risk (5.82, 0.05); protein (5.82, 0.05); incident gout (5.82, 0.05); prolong survival (5.82, 0.05); participant (5.82, 0.05); androgen deprivation therapy (5.82, 0.05); time (5.82, 0.05); particle size (5.82, 0.05); dietary (5.82, 0.05); fat (5.82, 0.05); chinese men and women (5.82, 0.05); nutrition (5.82, 0.05); inflammatory stress (5.82, 0.05); hepatocellular cancer (5.82, 0.05); dietary magnesium deficiency (5.82, 0.05); areca (5.82, 0.05); dietary surveys and nutritional epidemiology (5.82, 0.05); allopurinol (5.82, 0.05); survivor (5.82, 0.05); tumor necrosis factor (5.82, 0.05); primary care (5.82, 0.05); aging (5.82, 0.05); hdl cholesterol (5.82, 0.05); rats (5.82, 0.05); homa (5.82, 0.05); older adult (5.82, 0.05); macronutrient (5.82, 0.05); italy (5.82, 0.05); diagnosis (5.82, 0.05); serum and liver biomarkers (5.82, 0.05); colorectal cancer survivors (5.82, 0.05); clinical feature (5.82, 0.05); primary liver cancer (5.82, 0.05); symptom (5.82, 0.05); 25ohd (5.82, 0.05); ldl cholesterol (5.82, 0.05); diets (5.82, 0.05); metabolome (5.82, 0.05); interleukin 6 (5.82, 0.05); precancerous colorectal polyps (5.82, 0.05); diabetes mellitus (3.46, 0.1); colorectal neoplasms (3.35, 0.1); tyg index (3.16, 0.1); uric acid (3.16, 0.1); adenomas (3.16, 0.1); food frequency questionnaire (3.16, 0.1); inter‐ (3.16, 0.1); increased risk (3.16, 0.1); dietary carbohydrates (3.16, 0.1); chlorogenic acids (3.16, 0.1); hereditary tumors (3.16, 0.1); wheat bran (3.16, 0.1); body fat (3.16, 0.1); fap (3.16, 0.1); food-derived bioactive compounds (3.16, 0.1); individual variability (3.16, 0.1); ovarian cancer (3.16, 0.1); nucleotide polymorphisms (3.16, 0.1); high-fat diet (3.16, 0.1); polyphenols (3.16, 0.1); anthropometric measurements (3.16, 0.1); low-inflammatory diet (3.16, 0.1); cohort studies (2.89, 0.1); c-reactive protein (2.89, 0.1); prognosis (2.78, 0.1); colonoscopy (2.67, 0.5); survival (2.67, 0.5); adiponectin (2.56, 0.5); helicobacter pylori (2.22, 0.5); obesity-linked cancers (2.22, 0.5); cancer screening (2.22, 0.5); nhanes (2.22, 0.5); polyphenol (2.22, 0.5); obesity (2.05, 0.5); diabetes (1.99, 0.5); hypertension (1.99, 0.5); association (1.99, 0.5); insulin (1.88, 0.5); recurrence (1.77, 0.5) |
| 7 | 26 | 0.917 | 2015 | vitamin d | vitamin d (19.85, 1.0E-4); astaxanthin (6.6, 0.05); activated receptor gamma (6.6, 0.05); omega-3 (6.6, 0.05); nutrition transition (6.6, 0.05); visceral adiposity (6.6, 0.05); taurine (6.6, 0.05); multiethnic cohort (6.6, 0.05); apc (6.6, 0.05); metabolic switch (6.6, 0.05); preperitoneal fat thickness (6.6, 0.05); phospholipases (6.6, 0.05); mice (6.6, 0.05); underserved (6.6, 0.05); familial adenomatous polyposis (6.6, 0.05); dairy (6.6, 0.05); socioeconomic status (6.6, 0.05); sports medicine (6.6, 0.05); adiposity (6.6, 0.05); skeletal muscle mass (6.6, 0.05); fatty acid composition (6.6, 0.05); selenium (6.6, 0.05); proteases (6.6, 0.05); serum metabolites (6.6, 0.05); sustainable aquaculture (6.6, 0.05); factor kappa b (6.6, 0.05); ms (6.6, 0.05); ppar gamma (6.6, 0.05); metabonomics (6.6, 0.05); pancreatic cancer cells (6.6, 0.05); homeostasis (6.6, 0.05); 3h-quinazolin-4-one (6.6, 0.05); uplc-ms (6.6, 0.05); fish (6.6, 0.05); south korea (6.6, 0.05); folate intake (6.6, 0.05); mediating factors (6.6, 0.05); cell proliferation (6.6, 0.05); hif-1 alpha (6.6, 0.05); immune cell (6.6, 0.05); comorbidities (6.6, 0.05); preneoplastic lesions (6.6, 0.05); chemoprevention (5.87, 0.05); exercise (5.21, 0.05); colorectal neoplasia (4.66, 0.05); insulin resistance (4.16, 0.05); uric acid (3.9, 0.05); bmi change (3.9, 0.05); ppar ligand (3.9, 0.05); cancer survivors (3.9, 0.05); oxldl (3.9, 0.05); metabolic risk factors (3.9, 0.05); leiomyoma (3.9, 0.05); diet quality (3.9, 0.05); ahei (3.9, 0.05); precancerous polyps (3.9, 0.05); adipocytes (3.9, 0.05); waist circumference change (3.9, 0.05); mcc-555 (3.9, 0.05); thioredoxin (3.9, 0.05); glutaredoxin (3.9, 0.05); psychosocial (3.9, 0.05); azoxymethane (3.9, 0.05); sleep apnea obstructive (3.9, 0.05); aberrant crypt foci (3.9, 0.05); phytochemicals (3.9, 0.05); immunohistochemistty (3.9, 0.05); dash (3.9, 0.05); colorectal adenoma (3.47, 0.1); physical activity (3.15, 0.1); elderly (2.93, 0.1); autophagy (2.93, 0.1); tumor vasculature (2.93, 0.1); sarcopenia (2.87, 0.1); mortality (2.4, 0.5); diet patterns (2.33, 0.5); fruit (2.33, 0.5); pancreatic cancer (2.33, 0.5); carcinoembryonic antigen (2.33, 0.5); insulin receptor (2.33, 0.5); colorectal neoplasms (2.25, 0.5); body mass index (2.17, 0.5); advanced colorectal neoplasia (1.9, 0.5); prospective study (1.9, 0.5); lipid profile (1.9, 0.5); prognosis (1.87, 0.5); epidemiology (1.87, 0.5); colonoscopy (1.79, 0.5); survival (1.79, 0.5); adiponectin (1.72, 0.5); mediterranean diet (1.57, 0.5); diabetes (1.34, 0.5); diet (1.34, 0.5); breast cancer (1.34, 0.5); hypertension (1.34, 0.5); association (1.34, 0.5); biomarkers (1.31, 0.5); colon cancer (1.28, 0.5); insulin (1.26, 0.5); recurrence (1.19, 0.5) |
| 8 | 25 | 0.975 | 2016 | probiotics | probiotics (18.86, 1.0E-4); irritable bowel syndrome (15.51, 1.0E-4); dysbiosis (11.74, 0.001); prebiotics (11.74, 0.001); inflammatory bowel disease (8.91, 0.005); gut microbiome (8.91, 0.005); whole genome sequencing (7.74, 0.01); probiotic bacteria (7.74, 0.01); proliferation inhibition (7.74, 0.01); fecal microbiota transplantation (7.74, 0.01); applications (7.74, 0.01); interleukin 18 (7.74, 0.01); antibiotics (7.74, 0.01); functional foods (7.74, 0.01); immunomodulation (7.74, 0.01); respiratory disease (7.74, 0.01); intestinal epithelial cells (7.74, 0.01); mechanism of action (7.74, 0.01); urinary tract tumor (7.74, 0.01); adhesion (7.74, 0.01); modulation (7.74, 0.01); lactococcus lactis (7.74, 0.01); microbiota-mitochondria crosstalk (7.74, 0.01); multilevel model (7.74, 0.01); argentina (7.74, 0.01); gut microbiota (6.27, 0.05); systemic inflammatory response (5.01, 0.05); environment (5.01, 0.05); nuclear receptors (5.01, 0.05); aspirin use (5.01, 0.05); bifidobacterium (5.01, 0.05); 40s aged relative person (5.01, 0.05); family history (5.01, 0.05); liver disease (5.01, 0.05); health and disease (5.01, 0.05); therapy (5.01, 0.05); virus (5.01, 0.05); bowel disease (5.01, 0.05); microbial community (5.01, 0.05); lactobacillus (5.01, 0.05); viral database (5.01, 0.05); developing countries (5.01, 0.05); obesity (4.78, 0.05); ulcerative coliti (4.01, 0.05); intestinal microbiota (4.01, 0.05); metabolic syndrome (3.6, 0.1); risk prediction (3.37, 0.1); cardiovascular risk (3.37, 0.1); dietary patterns (3.37, 0.1); microbiome (2.91, 0.1); rectal cancer (2.01, 0.5); inflammation (1.44, 0.5); mortality (1.35, 0.5); diabetes mellitus (1.31, 0.5); colorectal neoplasms (1.27, 0.5); body mass index (1.22, 0.5); prognosis (1.05, 0.5); epidemiology (1.05, 0.5); colonoscopy (1.01, 0.5); survival (1.01, 0.5); adiponectin (0.97, 0.5); risk factor (0.92, 0.5); diabetes (0.75, 0.5); breast cancer (0.75, 0.5); hypertension (0.75, 0.5); association (0.75, 0.5); diet (0.73, 0.5); insulin (0.71, 0.5); recurrence (0.67, 0.5); risk factors (0.67, 0.5); colon (0.62, 0.5); colorectal neoplasm (0.62, 0.5); colorectal cancer (0.59, 0.5); cohort study (0.58, 0.5); risk (0.58, 0.5); sarcopenia (0.54, 0.5); meta-analysis (0.54, 0.5); screening (0.5, 0.5); dietary inflammatory index (0.5, 0.5); physical activity (0.5, 0.5); dyslipidemia (0.45, 1.0); leptin (0.45, 1.0); hyperinsulinemia (0.45, 1.0); metformin (0.37, 1.0); hepatocellular carcinoma (0.37, 1.0); prevalence (0.37, 1.0); the fiesta study (0.37, 1.0); colorectal polyps (0.37, 1.0); nafld (0.37, 1.0); cohort studies (0.37, 1.0); adenoma (0.37, 1.0); blood pressure (0.37, 1.0); c-reactive protein (0.37, 1.0); adipose tissue (0.33, 1.0); type 2 diabetes (0.33, 1.0); colorectal neoplasia (0.33, 1.0); cohort (0.33, 1.0); microbiota (0.33, 1.0); smoking (0.33, 1.0); non-alcoholic fatty liver disease (0.33, 1.0) |
| 9 | 25 | 0.913 | 2017 | prognosis | prognosis (24.59, 1.0E-4); digestive tract cancer (19.77, 1.0E-4); mortality (15.92, 1.0E-4); the fiesta study (15.24, 1.0E-4); fiesta study (9.85, 0.005); complication (8.19, 0.005); lipid derivative (8.19, 0.005); protease inhibitors (6.79, 0.01); liver surgery (6.79, 0.01); molecular biology (6.79, 0.01); steatohepatiti (6.79, 0.01); oncology (6.79, 0.01); histology (6.79, 0.01); the abo blood type (6.79, 0.01); colorectal metastase (6.79, 0.01); liver damage (6.79, 0.01); haart (6.79, 0.01); epidermal growth factor (6.79, 0.01); colorectal cancer metastases (6.79, 0.01); non nucleoside reverse transcriptase inhibitors (6.79, 0.01); hiv (6.79, 0.01); vascular endothelial growth factor (6.79, 0.01); oxaliplatin based chemotherapy (6.79, 0.01); hepatic steatosis (6.79, 0.01); prediabetes (6.79, 0.01); neoadjuvant chemotherapy (6.79, 0.01); medication (6.79, 0.01); major hepatectomy (6.79, 0.01); bone mineral density (6.79, 0.01); fasting glucose (6.79, 0.01); gastric cancer (5.56, 0.05); cancer (4.68, 0.05); hypoadiponectinemia (4.09, 0.05); stem cells (4.09, 0.05); cell signaling (4.09, 0.05); surgical site infection (4.09, 0.05); febrile episodes (4.09, 0.05); antipyretics (4.09, 0.05); wound infection (4.09, 0.05); abdominal surgery (4.09, 0.05); pathogenice (4.09, 0.05); mutations (4.09, 0.05); adipose (4.09, 0.05); esophageal squamous cell carcinoma (4.09, 0.05); coli (4.09, 0.05); metabolic risk score (4.09, 0.05); insulin resistance (3.78, 0.1); colorectal adenoma (3.15, 0.1); prognostic factors (3.11, 0.1); t2dm (3.11, 0.1); neutrophil-to-lymphocyte ratio (3.11, 0.1); platelet-to-lymphocyte ratio (3.11, 0.1); blood type (3.11, 0.1); postoperative outcomes (3.11, 0.1); surgery (2.5, 0.5); cvd (2.5, 0.5); complications (2.5, 0.5); prediction (2.5, 0.5); inflammation (2.32, 0.5); nash (2.06, 0.5); esophageal cancer (2.06, 0.5); colorectal neoplasms (2.04, 0.5); c reactive protein (1.73, 0.5); colonoscopy (1.63, 0.5); colorectal cancer (1.62, 0.5); adiponectin (1.56, 0.5); risk factor (1.55, 0.5); diabetes (1.21, 0.5); diet (1.21, 0.5); breast cancer (1.21, 0.5); insulin (1.15, 0.5); recurrence (1.08, 0.5); risk factors (1.08, 0.5); gut microbiota (1.06, 0.5); hepatocellular carcinoma (1.06, 0.5); nafld (1.06, 0.5); blood pressure (1.06, 0.5); obesity (1.03, 0.5); cohort study (0.94, 0.5); risk (0.94, 0.5); screening (0.8, 0.5); dietary inflammatory index (0.8, 0.5); physical activity (0.8, 0.5); dyslipidemia (0.73, 0.5); leptin (0.73, 0.5); hyperinsulinemia (0.73, 0.5); metformin (0.59, 0.5); prevalence (0.59, 0.5); colorectal polyps (0.59, 0.5); cohort studies (0.59, 0.5); adenoma (0.59, 0.5); c-reactive protein (0.59, 0.5); sarcopenia (0.56, 0.5); meta-analysis (0.56, 0.5); adipose tissue (0.53, 0.5); type 2 diabetes (0.53, 0.5); colorectal neoplasia (0.53, 0.5); rectal cancer (0.53, 0.5); cohort (0.53, 0.5); microbiota (0.53, 0.5) |
| 10 | 25 | 0.847 | 2015 | dietary inflammatory index | dietary inflammatory index (15.05, 0.001); dyslipidemia (11.39, 0.001); prospective (10.21, 0.005); cohort (10.01, 0.005); neoplasm (8.92, 0.005); cardiovascular disease (6.42, 0.05); insulin resistance (6.3, 0.05); vascular endothelial growth factor inhibitors (5.1, 0.05); induced inflammation (5.1, 0.05); blood (5.1, 0.05); osas (5.1, 0.05); chinese men (5.1, 0.05); cigarette smoke (5.1, 0.05); triglyceride glucose index (5.1, 0.05); adverse effect (5.1, 0.05); plco (5.1, 0.05); japanese men (5.1, 0.05); untargeted metabolomics (5.1, 0.05); hypercholesterolemia (5.1, 0.05); nonalcoholic fatty liver disease (nafld) (5.1, 0.05); uhplc-q-tof-ms (5.1, 0.05); cancer incidence (5.1, 0.05); dii (5.1, 0.05); chinese population (5.1, 0.05); extrahepatic complications (5.1, 0.05); atherogenic index of plasma (5.1, 0.05); pro-inflammatory diet (5.1, 0.05); weight change (5.1, 0.05); radical surgery (5.1, 0.05); life (5.1, 0.05); propensity score methods (5.1, 0.05); predictor (5.1, 0.05); squamous cell carcinoma (5.1, 0.05); blood pressure (4.84, 0.05); body mass index (4.69, 0.05); diabetes mellitus (4.26, 0.05); insulin (3.99, 0.05); waist circumference (3.97, 0.05); cardiovascular diseases (3.97, 0.05); colonoscopy (3.87, 0.05); adiponectin (3.7, 0.1); hypertension (3.64, 0.1); glucose (3.22, 0.1); lung cancer (3.22, 0.1); triglyceride (3.22, 0.1); risk factors (2.56, 0.5); insulin sensitivity (2.49, 0.5); cluster analysis (2.49, 0.5); food frequency questionnaire (2.49, 0.5); non-small cell lung cancer (2.49, 0.5); glucose metabolism (2.49, 0.5); head and neck (2.49, 0.5); oscc (2.49, 0.5); dietary antioxidant index (2.49, 0.5); adolescent girls (2.49, 0.5); mental health (2.49, 0.5); international diabetes federation (2.49, 0.5); invariance (2.49, 0.5); 2 deoxy d glucose (2.49, 0.5); targeting survivin (2.49, 0.5); dose (2.49, 0.5); ethnicity (2.49, 0.5); homeostasis model assessment (2.49, 0.5); colorectal tumor (2.49, 0.5); factor analysis (2.49, 0.5); japanese (2.49, 0.5); up regulation (2.49, 0.5); glycolysis (2.49, 0.5); cancer-specific survival (2.49, 0.5); oral cavity (2.49, 0.5); validation (2.49, 0.5); glucose tolerance abnormality (2.49, 0.5); hypertriglyceridemia (2.49, 0.5); colorectal malignant neoplasm (2.49, 0.5); diabetes risk reduction diet score (2.49, 0.5); skin tag (2.49, 0.5); dna polymerase kappa (2.49, 0.5); body fat percentage (2.49, 0.5); administrative data (2.49, 0.5); calorie intake (2.49, 0.5); hba1c (2.49, 0.5); inhibition (2.49, 0.5); thyroid neoplasms (2.49, 0.5); prostate lung colorectal and ovarian cancer screening trial (2.49, 0.5); response (2.49, 0.5); cohort study (2.23, 0.5); sarcopenia (2.07, 0.5); meta-analysis (2.07, 0.5); survival (2.04, 0.5); screening (1.9, 0.5); metformin (1.81, 0.5); cohort studies (1.81, 0.5); adenoma (1.81, 0.5); leptin (1.74, 0.5); egf (1.61, 0.5); interleukin-6 (1.61, 0.5); acrochordon (1.61, 0.5); overall survival (1.61, 0.5); calcium (1.61, 0.5); t2dm (1.61, 0.5) |
| 11 | 24 | 0.95 | 2016 | prevention | prevention (14.16, 0.001); stress (11.67, 0.001); alcohol consumption (11.67, 0.001); c reactive protein (9.54, 0.005); obesity (6.86, 0.01); fermentation (6.35, 0.05); complication (6.35, 0.05); insulin resistance (6.16, 0.05); score (5.83, 0.05); nk cells (5.83, 0.05); adolescence (5.83, 0.05); endproduct (5.83, 0.05); adolescent obesity (5.83, 0.05); childhood obesity (5.83, 0.05); american (5.83, 0.05); soluble receptor (5.83, 0.05); puberty (5.83, 0.05); myeloid suppressor cell (5.83, 0.05); pathogenesis (5.83, 0.05); adenoma recurrence (5.83, 0.05); fidarestat (5.83, 0.05); rage (5.83, 0.05); inflammatory biomarkers (5.83, 0.05); cardiometabolic disease (5.83, 0.05); metaflammation (5.83, 0.05); preoperative immunonutrition (5.83, 0.05); dietary fiber (5.83, 0.05); braf (5.83, 0.05); polyposis (5.83, 0.05); homocysteine (5.83, 0.05); acarbose (5.83, 0.05); management (5.83, 0.05); aldose reductase (5.83, 0.05); prospective population-based study (5.83, 0.05); empirical dietary inflammatory pattern (5.83, 0.05); childhood (5.83, 0.05); microsatellite instability (5.83, 0.05); longevity (5.83, 0.05); renin-angiotensin system (5.83, 0.05); metabolic factors (5.83, 0.05); apc min mice (5.83, 0.05); angiotensin-converting enzyme inhibitor (5.83, 0.05); genome wide association (5.83, 0.05); environment interaction (5.83, 0.05); cancer risk (3.85, 0.05); colon (3.83, 0.1); diabetes mellitus (3.44, 0.1); type 2 diabetes (3.34, 0.1); adenomas (3.17, 0.1); early-onset (3.17, 0.1); hypoadiponectinemia (3.17, 0.1); german part (3.17, 0.1); skeletal muscles (3.17, 0.1); surgical site infection (3.17, 0.1); hereditary tumors (3.17, 0.1); colon carcinogenesis (3.17, 0.1); joint-effect (3.17, 0.1); hyperplastic polyp (hp) (3.17, 0.1); fap (3.17, 0.1); iran (3.17, 0.1); relative validity (3.17, 0.1); international diabetes federation (3.17, 0.1); inflammatory markers (3.17, 0.1); therapy (3.17, 0.1); adult (3.17, 0.1); wound infection (3.17, 0.1); abdominal surgery (3.17, 0.1); recommendation (3.17, 0.1); validation (3.17, 0.1); adolescent health (3.17, 0.1); high-fat diet (3.17, 0.1); adipose (3.17, 0.1); non-alcoholic steatohepatitis (nash) (3.17, 0.1); low-inflammatory diet (3.17, 0.1); prognosis (2.77, 0.1); epidemiology (2.77, 0.1); colonoscopy (2.66, 0.5); adiponectin (2.54, 0.5); fatty liver (2.23, 0.5); reproducibility (2.23, 0.5); bacteria (2.23, 0.5); shift (2.23, 0.5); overall survival (2.23, 0.5); medicare (2.23, 0.5); marker (2.23, 0.5); quality of life (2.23, 0.5); kras (2.23, 0.5); colorectal cancer (crc) (2.23, 0.5); non-metastatic colorectal cancer (2.23, 0.5); chain fatty acid (2.23, 0.5); chemo-radiotherapy (2.23, 0.5); intestinal microbiome (2.23, 0.5); apolipoprotein b (2.23, 0.5); sleep duration (2.23, 0.5); polyphenol (2.23, 0.5); disease-free survival (2.23, 0.5); inflammation (2.05, 0.5); breast cancer (1.98, 0.5); insulin (1.87, 0.5); recurrence (1.76, 0.5) |
| 12 | 22 | 0.893 | 2016 | diet | diet (12.06, 0.001); fibrosis (11.12, 0.001); nafld (10.54, 0.005); coronary heart disease (10.22, 0.005); colorectal cancer (8.1, 0.005); atherosclerosis (7.64, 0.01); mortality (5.85, 0.05); cvd (5.83, 0.05); hcc (5.83, 0.05); acetaldehyde (5.55, 0.05); coffee consumption (5.55, 0.05); hypoxia (5.55, 0.05); gut-brain axis (5.55, 0.05); prostate cancer prevention (5.55, 0.05); tooth los (5.55, 0.05); virulence factors (5.55, 0.05); release (5.55, 0.05); secondary prevention (5.55, 0.05); natural history (5.55, 0.05); iodine intake (5.55, 0.05); protein intake (5.55, 0.05); carbohydrate intake (5.55, 0.05); macronutrients (5.55, 0.05); framingham risk score (5.55, 0.05); chronic kidney disease (5.55, 0.05); alcoholic dependence syndrome (5.55, 0.05); gastric bypas (5.55, 0.05); arterial disease (5.55, 0.05); levothyroxine (5.55, 0.05); prostate (5.55, 0.05); spred2 (5.55, 0.05); vitamin k (5.55, 0.05); decaffeinated coffee (5.55, 0.05); inflammatory marker (5.55, 0.05); body weight (5.55, 0.05); sam (5.55, 0.05); microrna-210 (5.55, 0.05); aldosterone (5.55, 0.05); alcoholic liver cirrhosis (5.55, 0.05); predisposing factors (5.55, 0.05); hemostasis (5.55, 0.05); human gut microbiota (5.55, 0.05); cardiovascular health (5.55, 0.05); primary prevention (5.55, 0.05); fat intake (5.55, 0.05); liver health (5.55, 0.05); menaquinones (5.55, 0.05); arachidonic acid (aa) (5.55, 0.05); cholangiocarcinoma (5.55, 0.05); linoleic acid (la) (5.55, 0.05); metagenome (5.55, 0.05); nafld fibrosis score (5.55, 0.05); nuts (5.55, 0.05); phylloquinone (5.55, 0.05); update (5.55, 0.05); body height (5.55, 0.05); predimed study (5.55, 0.05); thyroid stimulating hormone (5.55, 0.05); post-operative adhesions (5.55, 0.05); dietary glycemic load (5.55, 0.05); wheat aleurone (5.55, 0.05); oral glucose (5.55, 0.05); nash (4.77, 0.05); mediterranean diet (3.99, 0.05); health (3.38, 0.1); colonoscopy (3.06, 0.1); survival (3.06, 0.1); obesity (3.05, 0.1); metabolic syndrome (2.94, 0.1); adiponectin (2.93, 0.1); betaine (2.91, 0.1); oxldl (2.91, 0.1); german part (2.91, 0.1); bifidobacterium (2.91, 0.1); wcrf/aicr recommendations (2.91, 0.1); methyl donors (2.91, 0.1); wheat bran (2.91, 0.1); diet quality (2.91, 0.1); ahei (2.91, 0.1); relative validity (2.91, 0.1); health and disease (2.91, 0.1); cognitive impairment (2.91, 0.1); youden index (2.91, 0.1); periodontal disease (2.91, 0.1); virus (2.91, 0.1); chinese (2.91, 0.1); japanese (2.91, 0.1); colon cancer cells (2.91, 0.1); microbial community (2.91, 0.1); lactobacillus (2.91, 0.1); viral database (2.91, 0.1); cirrhosis (2.91, 0.1); psychosocial (2.91, 0.1); breast (2.91, 0.1); cardiovascular (2.91, 0.1); dna methylation (2.91, 0.1); thyroid neoplasms (2.91, 0.1); dash (2.91, 0.1); jun gene expression (2.91, 0.1); type 2 diabetes (2.89, 0.1) |
| 13 | 18 | 0.876 | 2016 | meta-analysis | meta-analysis (18.23, 1.0E-4); adipoq gene (16.93, 1.0E-4); adipoq (11.44, 0.001); er stress (8.44, 0.005); overall cancer (8.44, 0.005); fgf21 (8.44, 0.005); acinar cells (8.44, 0.005); summary rr (8.44, 0.005); blood-routine marker (8.44, 0.005); gcn2 (8.44, 0.005); genetic variation (8.44, 0.005); vegan diet (8.44, 0.005); site-specific cancer (8.44, 0.005); tnf-alpha (5.7, 0.05); genetic polymorphism (5.7, 0.05); inflammatory markers (5.7, 0.05); esophageal squamous cell carcinoma (5.7, 0.05); cancer (5.38, 0.05); nonalcoholic fatty liver (4.68, 0.05); pancreatic cancer (4.03, 0.05); polymorphism (4.03, 0.05); overweight (4.03, 0.05); adiponectin (3.94, 0.05); glucose (3.18, 0.1); incidence (3.18, 0.1); cancer risk (2.88, 0.1); the fiesta study (2.4, 0.5); insulin resistance (1.65, 0.5); cohort study (1.62, 0.5); colorectal neoplasm (1.51, 0.5); colorectal adenoma (1.37, 0.5); diabetes (1.22, 0.5); breast cancer (1.22, 0.5); colon cancer (1.19, 0.5); inflammation (1.01, 0.5); mortality (0.95, 0.5); diabetes mellitus (0.92, 0.5); colorectal neoplasms (0.89, 0.5); body mass index (0.86, 0.5); prognosis (0.75, 0.5); epidemiology (0.74, 0.5); colonoscopy (0.71, 0.5); survival (0.71, 0.5); risk factor (0.65, 0.5); diet (0.53, 0.5); hypertension (0.53, 0.5); association (0.53, 0.5); insulin (0.5, 0.5); recurrence (0.47, 0.5); risk factors (0.47, 0.5); colon (0.44, 1.0); risk (0.41, 1.0); metabolic syndrome (0.4, 1.0); sarcopenia (0.38, 1.0); screening (0.35, 1.0); dietary inflammatory index (0.35, 1.0); physical activity (0.35, 1.0); dyslipidemia (0.32, 1.0); leptin (0.32, 1.0); hyperinsulinemia (0.32, 1.0); obesity (0.31, 1.0); metformin (0.26, 1.0); gut microbiota (0.26, 1.0); hepatocellular carcinoma (0.26, 1.0); prevalence (0.26, 1.0); colorectal polyps (0.26, 1.0); nafld (0.26, 1.0); cohort studies (0.26, 1.0); adenoma (0.26, 1.0); blood pressure (0.26, 1.0); c-reactive protein (0.26, 1.0); adipose tissue (0.23, 1.0); type 2 diabetes (0.23, 1.0); colorectal neoplasia (0.23, 1.0); rectal cancer (0.23, 1.0); cohort (0.23, 1.0); microbiota (0.23, 1.0); smoking (0.23, 1.0); non-alcoholic fatty liver disease (0.23, 1.0); oxidative stress (0.23, 1.0); igf-1 (0.23, 1.0); atherosclerosis (0.2, 1.0); cardiovascular disease (0.2, 1.0); postmenopausal women (0.2, 1.0); exercise (0.2, 1.0); gastric cancer (0.2, 1.0); prevention (0.2, 1.0); cancer prevention (0.2, 1.0); adipocytokine (0.2, 1.0); health (0.2, 1.0); biomarkers (0.2, 1.0); digestive tract cancer (0.17, 1.0); age (0.17, 1.0); lung cancer (0.17, 1.0); cancer mortality (0.17, 1.0); colonic neoplasms (0.17, 1.0); triglyceride (0.17, 1.0); mediterranean diet (0.17, 1.0); metaanalysis (0.17, 1.0); proliferation (0.17, 1.0) |
| 14 | 15 | 0.971 | 2016 | colorectal cancer | colorectal cancer (27.18, 1.0E-4); obesity (9.65, 0.005); survival (9.33, 0.005); association (7.32, 0.01); incidence (6.65, 0.01); colon (6.07, 0.05); physical activity (4.83, 0.05); biomarker (4.19, 0.05); adjuvant chemotherapy (3.36, 0.1); apolipoproteins (3.36, 0.1); nutrition therapy (3.36, 0.1); cea (3.36, 0.1); individual screening (3.36, 0.1); apolipoprotein a-i (3.36, 0.1); dietary supplements (3.36, 0.1); molecular epidemiology (3.36, 0.1); asia-pacific region (3.36, 0.1); ampd2 (3.36, 0.1); bioelectrical impedance analysis (3.36, 0.1); environment and public health (3.36, 0.1); chronic renal failure (3.36, 0.1); gastric neoplasms (3.36, 0.1); mobile health telehealth (3.36, 0.1); lipid metabolism (3.36, 0.1); carcinoma (3.36, 0.1); follow-up programs (3.36, 0.1); adipose-derived stem cell (3.36, 0.1); incretin (3.36, 0.1); liver cancer (3.36, 0.1); hypothesis (3.36, 0.1); determinant (3.36, 0.1); long-term follow-up (3.36, 0.1); food variety (3.36, 0.1); asia-pacific colorectal screening scoring system (3.36, 0.1); hispanic (3.36, 0.1); global research trends (3.36, 0.1); platelet to lymphocyte ratio (3.36, 0.1); glucagon-like peptide-1 (3.36, 0.1); pentoxifylline (3.36, 0.1); adequate nutrition (3.36, 0.1); pattern (3.36, 0.1); laparoscopic colectomy (3.36, 0.1); health american association (3.36, 0.1); pathway analysis (3.36, 0.1); colorectal cancer: clinical research < gastroenterology (3.36, 0.1); hyperuricemia (3.36, 0.1); endoscopy (3.36, 0.1); data mining (3.36, 0.1); malaysia (3.36, 0.1); national institute (3.36, 0.1); liver neoplasms (3.36, 0.1); nodule (3.36, 0.1); gwas (3.36, 0.1); colorectal cancer: epidemiology and surveillance < gastroenterology (3.36, 0.1); mas (3.36, 0.1); acetyl-coa (3.36, 0.1); bone marrow suppression (3.36, 0.1); fecal hemoglobin concentration (3.36, 0.1); blood routine parameter (3.36, 0.1); migrants health (3.36, 0.1); endoscopy: colon < gastroenterology (3.36, 0.1); hand-foot syndrome (3.36, 0.1); digestive system neoplasm (3.36, 0.1); healthy (3.36, 0.1); epigenetics (3.36, 0.1); chemotherapy-related adverse effects (3.36, 0.1); oral premalignancy (3.36, 0.1); metabolic pathways (3.36, 0.1); health monitoring (3.36, 0.1); event (3.36, 0.1); angiotensin-ii (3.36, 0.1); bibliometric (3.36, 0.1); immunohistochemistry (3.36, 0.1); prognostic factors (3.32, 0.1); nhanes (3.32, 0.1); postoperative outcomes (3.32, 0.1); coronary artery calcification (3.32, 0.1); microbiota (3.18, 0.1); smoking (3.18, 0.1); cancer mortality (3.01, 0.1); polyps (3.01, 0.1); exercise (2.77, 0.1); gastric cancer (2.77, 0.1); prevention (2.77, 0.1); health (2.77, 0.1); cancer risk (2.77, 0.1); glucose (2.35, 0.5); lung cancer (2.35, 0.5); metaanalysis (2.35, 0.5); proliferation (2.35, 0.5); c reactive protein (2.35, 0.5); apoptosis (2.35, 0.5); prostate cancer (2.35, 0.5); colon cancer (2.3, 0.5); colorectal neoplasms (2.29, 0.5); risk factor (2.21, 0.5); screening colonoscopy (2.01, 0.5); oral cancer (2.01, 0.5); sex difference (2.01, 0.5); computed tomography (2.01, 0.5) |
| 15 | 14 | 0.931 | 2017 | cancer | cancer (30.52, 1.0E-4); lung cancer (12.87, 0.001); colorectal cancer (10.37, 0.005); obese (10.07, 0.005); breast cancer (9.38, 0.005); hyperinsulinemia (6.92, 0.01); colon cancer (6.74, 0.01); metabolic abnormalities (6.42, 0.05); soy isoflavonoids (5.03, 0.05); sirtuin 6 (5.03, 0.05); pernicious anemia (5.03, 0.05); beta blocker (5.03, 0.05); obesity phenotypes (5.03, 0.05); rifaximin (5.03, 0.05); mouse (5.03, 0.05); health disparities (5.03, 0.05); central nervous system (5.03, 0.05); korean diet (5.03, 0.05); cerebellar ataxia (5.03, 0.05); metabolically benign (5.03, 0.05); self defense official (5.03, 0.05); depression (5.03, 0.05); white blood cell count (5.03, 0.05); toxic encephalopathy (5.03, 0.05); rectosigmoid adenoma (5.03, 0.05); inflammatory (5.03, 0.05); migraine (5.03, 0.05); fluoropyrimides (5.03, 0.05); duration (5.03, 0.05); metachronous colorectal neoplasia (5.03, 0.05); pressure (5.03, 0.05); endometriosis (5.03, 0.05); meat and milk (5.03, 0.05); morbid obesity (5.03, 0.05); style factor (5.03, 0.05); adults (5.03, 0.05); quantitative fecal immunochemical test (5.03, 0.05); risk stratification (5.03, 0.05); igf 1 receptor (5.03, 0.05); resistance (5.03, 0.05); bioelectrical impedance analyses (5.03, 0.05); apnea (5.03, 0.05); pet/ct (5.03, 0.05); cross-sectional studies (5.03, 0.05); inhibitor (5.03, 0.05); intermittent hypoxia (5.03, 0.05); periodontal probing depth (5.03, 0.05); united states (4.86, 0.05); overweight (4.86, 0.05); body mass index (4.86, 0.05); prognosis (4.18, 0.05); survival (4.01, 0.05); population (3.85, 0.05); adiponectin (3.84, 0.1); glucose (3.11, 0.1); cancer mortality (3.11, 0.1); apoptosis (3.11, 0.1); expression (3.11, 0.1); colorectal adenoma (3.01, 0.1); recurrence (2.65, 0.5); postmenopausal women (2.54, 0.5); exercise (2.54, 0.5); cancer prevention (2.54, 0.5); colorectal neoplasm (2.48, 0.5); insulin sensitivity (2.43, 0.5); glucose metabolism (2.43, 0.5); cell (2.43, 0.5); linoleic acid (2.43, 0.5); longitudinal study (2.43, 0.5); increased risk (2.43, 0.5); alpha-fetoprotein (2.43, 0.5); wcrf/aicr recommendations (2.43, 0.5); tumor (2.43, 0.5); pentamethylquercetin (2.43, 0.5); monosodium glutamate mice (2.43, 0.5); schizophrenia (2.43, 0.5); periodontal disease (2.43, 0.5); dose (2.43, 0.5); standardised incidence ratio (sir) (2.43, 0.5); weight gain (2.43, 0.5); homeostasis model assessment (2.43, 0.5); anxiety (2.43, 0.5); up regulation (2.43, 0.5); occult blood (2.43, 0.5); glucose tolerance abnormality (2.43, 0.5); noninvasive fibrosis score (2.43, 0.5); liver steatosis (2.43, 0.5); diabetes risk reduction diet score (2.43, 0.5); synchronizer (2.43, 0.5); thioredoxin (2.43, 0.5); glutaredoxin (2.43, 0.5); dna polymerase kappa (2.43, 0.5); incidence rate (2.43, 0.5); mediation analysis (2.43, 0.5); nonobesity (2.43, 0.5); hydroxyoctadecadienoic acids (2.43, 0.5); immunohistochemistty (2.43, 0.5); prostate lung colorectal and ovarian cancer screening trial (2.43, 0.5); malignant neoplastic disorders (2.43, 0.5); response (2.43, 0.5) |
| 16 | 12 | 0.876 | 2017 | colonoscopy | colonoscopy (26.05, 1.0E-4); smoking (16.82, 1.0E-4); non-alcoholic fatty liver disease (10.45, 0.005); guideline (10.04, 0.005); prevalence (9.64, 0.005); advanced adenoma (8.38, 0.005); fecal immunochemical test (7.26, 0.01); alanine aminotransferase (6.89, 0.01); young population (6.89, 0.01); brca2 mutation (6.89, 0.01); risk score (6.89, 0.01); occult blood test (6.89, 0.01); sex (6.89, 0.01); hemoglobin (6.89, 0.01); estrogen plus progestin (6.89, 0.01); performance (6.89, 0.01); colorectal polyps (4.71, 0.05); adenoma (4.71, 0.05); gastric polyps (4.18, 0.05); machine learning (4.18, 0.05); health check (4.18, 0.05); society task force (4.18, 0.05); skeletal muscles (4.18, 0.05); gallbladder polyps (4.18, 0.05); advanced neoplasia (4.18, 0.05); schizophrenia (4.18, 0.05); artificial intelligence (4.18, 0.05); standardised incidence ratio (sir) (4.18, 0.05); fatty liver index (4.18, 0.05); regression (4.18, 0.05); young adults (4.18, 0.05); breast (4.18, 0.05); gastroscopy (4.18, 0.05); sleep apnea obstructive (4.18, 0.05); malignant neoplastic disorders (4.18, 0.05); sarcopenia (3.34, 0.1); cancer screening (3.2, 0.1); gastric neoplasm (3.2, 0.1); type 2 diabetes mellitus (3.2, 0.1); endoscopic submucosal dissection (3.2, 0.1); colorectal neoplasm (2.85, 0.1); obesity (2.71, 0.1); recurrence (2.63, 0.5); liver fibrosis (2.58, 0.5); hcc (2.58, 0.5); carcinoembryonic antigen (2.58, 0.5); prediction (2.58, 0.5); association (2.26, 0.5); inflammation (2.21, 0.5); advanced colorectal neoplasia (2.14, 0.5); population (2.14, 0.5); inflammatory bowel disease (2.14, 0.5); body mass index (1.88, 0.5); age (1.8, 0.5); metaanalysis (1.8, 0.5); prognosis (1.62, 0.5); epidemiology (1.62, 0.5); survival (1.55, 0.5); gastric cancer (1.53, 0.5); adiponectin (1.49, 0.5); risk factor (1.42, 0.5); colorectal adenoma (1.28, 0.5); diabetes (1.16, 0.5); diet (1.16, 0.5); hypertension (1.16, 0.5); cohort studies (1.13, 0.5); insulin (1.09, 0.5); risk factors (1.03, 0.5); colon (0.96, 0.5); colorectal neoplasms (0.9, 0.5); cohort study (0.89, 0.5); risk (0.89, 0.5); hyperinsulinemia (0.83, 0.5); meta-analysis (0.83, 0.5); diabetes mellitus (0.83, 0.5); dietary inflammatory index (0.76, 0.5); physical activity (0.76, 0.5); screening (0.72, 0.5); dyslipidemia (0.7, 0.5); leptin (0.7, 0.5); metformin (0.57, 0.5); gut microbiota (0.57, 0.5); hepatocellular carcinoma (0.57, 0.5); the fiesta study (0.57, 0.5); nafld (0.57, 0.5); blood pressure (0.57, 0.5); c-reactive protein (0.57, 0.5); adipose tissue (0.5, 0.5); type 2 diabetes (0.5, 0.5); colorectal neoplasia (0.5, 0.5); rectal cancer (0.5, 0.5); cohort (0.5, 0.5); microbiota (0.5, 0.5); oxidative stress (0.5, 0.5); igf-1 (0.5, 0.5); atherosclerosis (0.44, 1.0); cardiovascular disease (0.44, 1.0); postmenopausal women (0.44, 1.0); exercise (0.44, 1.0); prevention (0.44, 1.0) |
|  |  |  |  |  |  |

# 10.Keywords

| **Keywords ranked by frequency and centrality** | | | | |
| --- | --- | --- | --- | --- |
| **Rank** | **Keywords** | **Frequency** | **Keywords** | **Centrality** |
| 1 | metabolic syndrome | 413 | inflammation | 0.19 |
| 2 | colorectal cancer | 292 | prevalence | 0.12 |
| 3 | risk | 230 | physical activity | 0.11 |
| 4 | obesity | 147 | survival | 0.1 |
| 5 | association | 119 | colon | 0.09 |
| 6 | cancer | 115 | disease | 0.09 |
| 7 | insulin resistance | 108 | adipose tissue | 0.09 |
| 8 | colon cancer | 94 | fatty liver disease | 0.09 |
| 9 | colorectal adenoma | 77 | smoking | 0.09 |
| 10 | mortality | 76 | mortality | 0.08 |
| 11 | body mass index | 74 | cardiovascular disease | 0.08 |
| 12 | inflammation | 66 | prostate cancer | 0.08 |
| 13 | metaanalysis | 57 | health | 0.08 |
| 14 | prevalence | 51 | cohort | 0.08 |
| 15 | breast cancer | 51 | diet | 0.08 |
| 16 | survival | 49 | expression | 0.07 |
| 17 | colon | 43 | colonoscopy | 0.07 |
| 18 | physical activity | 42 | glucose | 0.07 |
| 19 | cardiovascular disease | 41 | cohort study | 0.07 |
| 20 | disease | 39 | oxidative stress | 0.07 |
| 21 | diabetes mellitus | 34 | insulin resistance | 0.06 |
| 22 | colorectal neoplasm | 31 | cancer risk | 0.06 |
| 23 | adipose tissue | 29 | population | 0.06 |
| 24 | component | 28 | impact | 0.06 |
| 25 | adiponectin | 28 | prevention | 0.06 |
| 26 | prostate cancer | 27 | plasma adiponectin | 0.06 |
| 27 | expression | 27 | metabolism | 0.06 |
| 28 | c reactive protein | 27 | body mass index | 0.05 |
| 29 | health | 26 | metaanalysis | 0.05 |
| 30 | prognosis | 26 | breast cancer | 0.05 |
| 31 | insulin | 26 | prognosis | 0.05 |
| 32 | cohort | 25 | insulin | 0.05 |
| 33 | colonoscopy | 25 | rectal cancer | 0.05 |
| 34 | cancer risk | 24 | hypertension | 0.05 |
| 35 | diet | 22 | colorectal neoplasm | 0.04 |
| 36 | rectal cancer | 21 | component | 0.04 |
| 37 | population | 19 | adiponectin | 0.04 |
| 38 | hepatocellular carcinoma | 19 | c reactive protein | 0.04 |
| 39 | recurrence | 19 | hepatocellular carcinoma | 0.04 |
| 40 | glucose | 18 | united states | 0.04 |
| 41 | impact | 17 | metabolic syndrome | 0.03 |
| 42 | united states | 17 | association | 0.03 |
| 43 | epidemiology | 17 | cancer | 0.03 |
| 44 | cohort study | 16 | colon cancer | 0.03 |
| 45 | hypertension | 16 | colorectal adenoma | 0.03 |
| 46 | oxidative stress | 14 | diabetes mellitus | 0.03 |
| 47 | biomarker | 14 | epidemiology | 0.03 |
| 48 | postmenopausal women | 14 | biomarker | 0.03 |
| 49 | fatty liver disease | 13 | adenomatous polyp | 0.03 |
| 50 | prevention | 13 | polyp | 0.03 |
| 51 | adenomatous polyp | 12 | polymorphism | 0.03 |
| 52 | polyp | 12 | gastric cancer | 0.03 |
| 53 | pancreatic cancer | 12 | growth | 0.03 |
| 54 | cigarette smoking | 12 | adenocarcinoma | 0.03 |
| 55 | smoking | 11 | recurrence | 0.02 |
| 56 | plasma adiponectin | 11 | postmenopausal women | 0.02 |
| 57 | polymorphism | 11 | pancreatic cancer | 0.02 |
| 58 | gastric cancer | 11 | growth factor i | 0.02 |
| 59 | growth factor i | 11 | women | 0.02 |
| 60 | women | 11 | coronary heart disease | 0.02 |
| 61 | marker | 11 | risk | 0.01 |
| 62 | blood pressure | 11 | obesity | 0.01 |
| 63 | metabolism | 10 | cigarette smoking | 0.01 |
| 64 | growth | 10 | marker | 0.01 |
| 65 | adenocarcinoma | 10 | blood pressure | 0.01 |
| 66 | coronary heart disease | 10 | leptin | 0.01 |
| 67 | leptin | 10 | cell | 0.01 |
| 68 | cell | 10 | colorectal cancer | 0 |

# 11.new reference

**New literature on colorectal cancer and metabolic syndrome published in 2023**

| **ID** | **Title** | **Citations** |
| --- | --- | --- |
| 1 | systematic review and meta-analysis of the impact of bariatric surgery on future cancer risk | 6 |
| 2 | connecting the dots in the associations between diet, obesity, cancer, and micrornas | 3 |
| 3 | molecular mechanisms of western diet-induced obesity and obesity-related carcinogenesis-a narrative review | 2 |
| 4 | the emerging role of pi3k inhibitors for solid tumour treatment and beyond | 2 |
| 5 | high-fat diet and estrogen modulate the gut microbiota in a sex-dependent manner in mice | 2 |
| 6 | prognostic nomograms integrating preoperative serum lipid derivative and systemic inflammatory marker of patients with non-metastatic colorectal cancer undergoing curative resection | 1 |
| 7 | associations of cholecystectomy with the risk of colorectal cancer: a mendelian randomization study | 1 |
| 8 | carbohydrate quality indices and colorectal cancer risk: a case-control study | 1 |
| 9 | new insights into functional cereal foods as an alternative for dairy products: a review | 1 |
| 10 | association between serum carcinoembryonic antigen and cardiometabolic risks: implication for cardiometabolic prevention | 0 |
| 11 | deficiency of angiopoietin-like 4 enhances cd8(+) t cell bioactivity via metabolic reprogramming for impairing tumour progression | 0 |
| 12 | the composition of small extracellular vesicles (sevs) in the blood plasma of colorectal cancer patients reflects the presence of metabolic syndrome and correlates with angiogenesis and the effectiveness of thermoradiation therapy | 0 |
| 13 | new horizons: epidemiology of obesity, diabetes mellitus, and cancer prognosis | 0 |
| 14 | the effect of metabolic syndrome on postoperative complications and long-term survival of patients with colorectal cancer | 0 |
| 15 | risk factors for advanced colorectal neoplasm in young adults: a meta-analysis | 0 |
| 16 | prevalence of clinical signs, symptoms and comorbidities at diagnosis of acromegaly: a systematic review in accordance with prisma guidelines | 0 |
| 17 | complete metabolic response to combined immune checkpoint inhibition after progression of metastatic colorectal cancer on pembrolizumab: a case report | 0 |
| 18 | a critical perspective on the supplementation of akkermansia muciniphila: benefits and harms | 0 |
| 19 | autophagy and the insulin-like growth factor (igf) system in colonic cells: implications for colorectal neoplasia | 0 |
| 20 | prediagnostic serum glyceraldehyde-derived advanced glycation end products and mortality among colorectal cancer patients | 0 |
| 21 | risk of colorectal adenocarcinoma in men receiving androgen deprivation therapy for prostate cancer; a nationwide cohort study | 0 |
| 22 | association between metabolic syndrome and participation in colorectal cancer screening in japan: a retrospective cohort analysis using life study data | 0 |
| 23 | association between metabolic syndrome and gastric cancer risk: results from the health examinees study | 0 |
| 24 | novel biomarkers for inflammatory bowel disease and colorectal cancer: an interplay between metabolic dysregulation and excessive inflammation | 0 |
| 25 | the apolipoprotein b and apolipoprotein a-i ratio serves as a strong prognostic factor for the overall survival of patients with colorectal cancer | 0 |
| 26 | alteration of metabolic syndrome is associated with the decreased risk of colorectal cancer | 0 |
| 27 | team-based approach to reduce malignancies in people with diabetes and obesity | 0 |
| 28 | association of modifiable lifestyle with colorectal cancer incidence and mortality according to metabolic status: prospective cohort study | 0 |
| 29 | the evidence surrounding non-alcoholic fatty liver disease in individuals with cancer: a systematic literature review | 0 |
| 30 | a narrative review on the use of probiotics in several diseases. evidence and perspectives | 0 |
| 31 | serum lipid profiles and cholesterol-lowering medication use in relation to subsequent risk of colorectal cancer in the uk biobank cohort | 0 |
| 32 | prospective analysis reveals associations between carbohydrate intakes, genetic predictors of short-chain fatty acid synthesis, and colorectal cancer risk | 0 |
| 33 | network analysis and nomogram in the novel classification and prognosis prediction of advanced schistosomiasis japonica | 0 |
| 34 | a scoring method to prioritize fecal occult blood testing as a first step in colorectal cancer screening in resource-limited settings | 0 |
| 35 | field synopsis of environmental and genetic risk factors of sporadic early-onset colorectal cancer and advanced adenoma | 0 |
| 36 | tnf-alpha-1031t/c gene polymorphism as a predictor of malnutrition in patients with gastric cancer | 0 |
| 37 | increased & alpha;-hb links colorectal cancer and diabetes by potentiating nf-& kappa;b signaling | 0 |
| 38 | the association between educational status and colorectal neoplasia: results from a screening cohort | 0 |
| 39 | association of 25(oh)-vitamin d and metabolic factors with colorectal polyps | 0 |
| 40 | regorafenib monotherapy as second-line treatment of patients with ras-mutant advanced colorectal cancer (stream): an academic, multicenter, single-arm, two-stage, phase ii study | 0 |
| 41 | the modulation of adipokines, adipomyokines, and sleep disorders on carcinogenesis | 0 |
| 42 | changing epidemiology of colorectal cancer - birth cohort effects and emerging risk factors | 0 |
| 43 | sleep and cancer recurrence and survival in patients with resected stage iii colon cancer: findings from calgb/swog 80702 (alliance) | 0 |
| 44 | risk factors for early-onset colorectal cancer: systematic review and meta-analysis | 0 |
| 45 | in vitro digestion of peanut skin releases bioactive compounds and increases cancer cell toxicity | 0 |
| 46 | high lymphocyte count as a significant risk factor for incisional hernia after laparoscopic colorectal surgery | 0 |
| 47 | emerging paradigms in inflammatory disease management: exploring bioactive compounds and the gut microbiota | 0 |
| 48 | a high-fat, high-cholesterol diet promotes intestinal inflammation by exacerbating gut microbiome dysbiosis and bile acid disorders in cholecystectomy | 0 |
| 49 | positive feedback loop between dietary nitrate intake and oral health | 0 |
| 50 | comorbid conditions associated with alopecia areata: a systematic review and meta-analysis | 0 |
| 51 | gut bacteria influence blastocystis sp. phenotypes and may trigger pathogenicity | 0 |
